# Cancer Risk Following Smoking Cessation in Korea

**DOI:** 10.1001/jamanetworkopen.2023.54958

**Published:** 2024-02-06

**Authors:** Eunjung Park, Hee-Yeon Kang, Min Kyung Lim, Byungmi Kim, Jin-Kyoung Oh

**Affiliations:** 1 Department of Cancer Control and Population Health, National Cancer Center Graduate School of Cancer Science and Policy, Goyang, Republic of Korea; 2 Division of Cancer Prevention, National Cancer Control Institute, National Cancer Center, Goyang, Republic of Korea; 3 Department of Social and Preventive Medicine, Inha University College of Medicine, Incheon, Republic of Korea

## Abstract

**Importance:**

Tobacco smoking is associated with increased risk of various cancers, and smoking cessation has been associated with reduced cancer risks, but it is still unclear how many years of smoking cessation are required to significantly reduce the cancer risk. Therefore, investigating the association of smoking cessation with cancer is essential.

**Objective:**

To investigate the time course of cancer risk according to the time elapsed since smoking cessation and the benefits of smoking cessation according to the age at quitting.

**Design, Setting, and Participants:**

This population-based, retrospective cohort study included Korean participants aged 30 years and older who underwent 2 or more consecutive health examinations under the National Health Insurance Service since 2002 and were followed-up until 2019. Data analysis was performed from April to September 2023.

**Exposures:**

Exposures included (1) time-updated smoking status based on biennial changes in smoking status, defined as complete quitters, transient quitters, relapsed quitters, continuous smokers, and never smokers; (2) duration of smoking cessation, defined as years since quitting; and (3) categorical variable for age at quitting.

**Main Outcomes and Measures:**

The primary cancer was ascertained using the cancer registry data: all-site cancer (*International Statistical Classification of Diseases and Related Health Problems, Tenth Revision [ICD-10]* codes C00-43, C45-96, or D45-D47), lung cancer (*ICD-10* code C34), liver cancer (*ICD-10* code C22), stomach cancer (*ICD-10* code C16), and colorectal cancer (*ICD-10* codes C18-20). Hazard ratios (HRs) and 95% CIs were estimated using a Cox proportional hazards regression model with follow-up years as the timescale.

**Results:**

Of the 2 974 820 participants, 1 727 340 (58.1%) were men (mean [SD] age, 43.1 [10.0] years), and 1 247 480 (41.9%) were women (mean [SD] age, 48.5 [9.9] years). Over a mean (SD) follow-up of 13.4 (0.1) years, 196 829 cancer cases were confirmed. Compared with continuous smokers, complete quitters had a lower risk of cancer, with HRs of 0.83 (95% CI, 0.80-0.86) for all cancer sites, 0.58 (95% CI, 0.53-0.62) for lung, 0.73 (95% CI, 0.64-0.82) for liver, 0.86 (95% CI, 0.79-0.93) for stomach, and 0.80 (95% CI, 0.72-0.89) for colorectum. The cancer risk exhibited a slightly higher value for 10 years after quitting compared with continued smoking and then it decreased over time, reaching 50% of the risk associated with continued smoking after 15 or more years. Lung cancer risk decreased 3 years earlier than that of other cancer types, with a larger relative reduction. Regardless of quitting age, a significant reduction in the cancer risk was observed. Quitting before the age of 50 years was associated with a greater reduction in lung cancer risk (HR, 0.43; 95% CI, 0.35-0.53) compared with quitting at age 50 years or later (HR, 0.61; 95% CI, 0.56-0.66).

**Conclusions and Relevance:**

In this population-based retrospective cohort study, sustained smoking cessation was associated with significantly reduced risk of cancer after 10 years since quitting. Quitting at any age helped reduce the cancer risk, and especially for lung cancer, early cessation before middle age exhibited a substantial risk reduction.

## Key Points

**Question:**

What duration of smoking cessation is associated with reduced risk of all-site, lung, liver, stomach, and colorectal cancers?

**Findings:**

In this population-based cohort study of more than 2 million participants, the cancer risk showed a slightly higher value for 10 years after quitting compared with continued smoking, and then gradually decreased, reaching 50% of the risk associated with continued smoking after 15 years or more. Lung cancer risk decreased 3 years earlier than that of other cancer types, with a larger relative reduction.

**Meaning:**

These findings suggest that sustained smoking cessation is associated with reduced risk of cancer, especially lung cancer, after 10 years since quitting smoking.

## Teaser

This Korean cohort study investigates the time course of cancer risk according to the time elapsed since smoking cessation and the benefits of smoking cessation according to the age at quitting.

## Introduction

Tobacco smoking is an avoidable risk factor for cancers.^1,2,3,4^ According to an International Agency for Research on Cancer monograph,^5^ smoking is a known causative factor in cancer development at multiple sites, including the lungs, stomach, colorectum, liver, pancreas, urinary bladder, and kidney. Several studies have reported that smoking cessation is associated with substantially reduced risk of all cancers^6^ and smoking-related cancers,^6,7,8^ such as lung^9,10^ esophageal,^11,12^ and laryngeal cancer.^13,14^

However, there is insufficient evidence on whether smoking cessation prevents other cancer types except for those mentioned above.^15^ Moreover, evidence on the benefits of smoking cessation has been primarily demonstrated in Western populations, which may differ from that reported in the general population owing to sociodemographic factors, disease prevalence, and smoking rates.^16,17^ In Korea, there is a sex disparity in tobacco use prevalence, with 40% to 50% of men and 4% to 8% of women using tobacco.^16^ Furthermore, it is still unclear how many years of smoking cessation are required to significantly reduce the cancer risk. Recent studies^18,19,20^ have suggested that the age at smoking cessation also plays a critical role in reducing the cancer risk. Therefore, investigating the association of the age of smoking cessation with cancer is essential. In this study, we investigated the time course of cancer risk according to the time elapsed since smoking cessation and the benefits of smoking cessation according to the age at quitting using longitudinal and repeated assessments of the smoking status using the nationally representative claims data obtained from the Korean population.

## Methods

### Study Design

We used health insurance claims data from the National Health Insurance Service (NHIS) collected between January 2002 and December 2017 in South Korea. The NHIS is a single mandatory national health insurance that covers Korea’s entire population (97% insured and 3% medical aid beneficiaries) and provides a general health screening program to all beneficiaries every 2 years.^21^ For this population-based retrospective cohort study, we enrolled 7 536 882 participants aged 30 years and older who underwent health screening during the initial examination cycle conducted between 2002 and 2003, which was set as the baseline period. Subsequently, these data were linked to the cancer registry data acquired from the Korea Central Cancer Registry and death records obtained from Statistics Korea. The rate of participation in health screening was 43.2% in 2002^22^ and 48.0% in 2003,^23^ and participants who underwent health screening were different in income status, employment status, region of residence, and disability status compared with the entire Korean population.^24^ A detailed description of the data collection process has been presented elsewhere.^25^

This study was approved by the institutional review board of the National Cancer Center, Korea. The study was conducted in accordance with the principles of the Declaration of Helsinki.^26^ The requirement for informed consent was waived because this study used anonymized secondary data. The study followed the Strengthening the Reporting of Observational Studies in Epidemiology (STROBE) reporting guideline.

### Study Participants

For the present analysis, we excluded participants with cancer or those who died before January 2006. We also excluded participants who reported being past smokers and who did not provide smoking information in 2 or more consecutive cycles or completed only the baseline questionnaire. Furthermore, participants without information on smoking duration, daily amount of smoking, income level, body mass index (BMI; calculated as weight in kilograms divided by height in meters squared), alcohol consumption, or physical activity, during the baseline were excluded (eAppendix and eFigure 1 in Supplement 1).

### Exposures

#### Time-Updated Smoking Status

Time-updated smoking status was defined using self-reported smoking information collected from health examinations biennially from 2002 to 2017. Participants were classified into 5 categories on the basis of biennial changes in their smoking behaviors until the occurrence of study outcomes: complete quitters, transient quitters, relapsed quitters, continuous smokers, and never smokers. In each 2-year examination cycle, quitters were defined as participants who reported being smokers in the previous cycle but were past smokers in the current cycle and were subsequently categorized into 3 mutually exclusive groups according to their smoking behaviors in the following cycles: complete quitters were defined as participants who reported being past smokers in all subsequent cycles after initially quitting, transient quitters were defined as those who alternated between reporting being current smokers and past smokers in subsequent cycles, and relapsed quitters were defined as those who reported smoking again in subsequent cycles. Continuous smokers and never smokers were defined as those who reported that they were current smokers and had never smoked, respectively, in every 2-year cycle (eFigure 2 in Supplement 1). In cases where smoking status was unavailable, we imputed missing data using assessments from the immediately preceding cycle (28.3% of participants) (eAppendix and eTable 1 in Supplement 1).

#### Duration of Smoking Cessation and Age at Quitting

To determine the duration of smoking cessation for all quitters, we calculated the time from the start of quitting to the end of the follow-up period and excluded the duration of smoking relapse. A categorical variable was created for age at quitting (quitting at the age of <50 years and ≥50 years), with continuous smokers as the reference group.

### Study Outcomes

The primary cancer was ascertained using the cancer registry data, covering the period from January 1, 2006, to December 31, 2019. In this study, all cancer cases were identified by the *International Statistical Classification of Diseases and Related Health Problems, Tenth Revision* (*ICD-10*) codes, specifically C00-C96 or D45-D47, except melanoma (*ICD-10 *code C44). Lung, liver, stomach, and colorectal cancers were defined as follows: *ICD-10 *code C34, lung cancer; *ICD-10 *code C22, liver cancer; *ICD-10 *code C16, stomach cancer; and *ICD-10 *codes C18 to C20, colorectal cancer. Cancer incidence rates are shown in eFigure 3 and eFigure 4 in Supplement 1.

### Statistical Analysis

 Data analysis was performed from April to September 2023. The follow-up for the study cohort started on January 1, 2006, and conducted until the first cancer event, cancer death, or right-censoring, whichever came first. Right-censoring occurred at the earliest of diagnosis of any cancer (except melanoma), a noncancerous death, or the end of the study period (December 31, 2019). Descriptive statistics were calculated to examine the general characteristics of the participants at baseline with stratification by sex and time-updated smoking status. Hazard ratios (HRs) and 95% CIs were estimated using a Cox proportional hazards regression model with follow-up years as the timescale. Crude, age-adjusted, and multivariable-adjusted HRs (aHRs) were estimated with stratification by sex. The aHRs were adjusted for age, pack-years, BMI, income level, physical activity, alcohol consumption, and presence or absence of chronic viral hepatitis and liver cirrhosis (only for the liver cancer analysis). Details on covariates are described in the eAppendix in Supplement 1. We did not detect any evidence indicating a violation of the proportional-hazards assumption for any exposure-outcome associations (eAppendix in Supplement 1).

Time course of cancer risk according to years since quitting was analyzed only in men in 2 ways. First, a categorical variable was created for years since quitting (<10, 10 to <15, and ≥15 years), with continuous smokers as the reference group, and the aHRs were estimated using the Cox proportional hazards regression analysis. Second, years since quitting were modeled as a continuous variable (up to 16 years), assigning continuous smokers a value of 0. Never smokers were assigned a value of 30, well above that for all quitters. In the Cox proportional hazards regression model, restricted cubic spline function was used for the variable of continuous years since quitting, as presented graphically.

Furthermore, to identify the association between the age at quitting and the cancer risk only in men, the aHRs were estimated using the Cox proportional hazards regression analysis. Because of the lack of cancer cases among women, we could not conduct further analyses to examine the associations among cancer risk, years since quitting, and age at quitting in women.

To ensure the stability and reliability of the findings, sensitivity analysis was conducted using a subset of the parent data (eFigure 5 in Supplement 1). The duration of smoking cessation was obtained from a questionnaire administered to past smokers (eFigure 6 in Supplement 1). Detailed descriptions are presented in eFigure 5 and the eAppendix in Supplement 1.

All statistical analyses were performed using the PHREG procedures in SAS Enterprise Guide version 7.1 (SAS Institute). All statistical tests were 2-sided, with a significance level of *P* < .05.

## Results

### Characteristics of Study Participants

A total of 2 974 820 participants were included in this cohort study (1 727 340 men [58.1%]; mean [SD] age, 43.1 [10.0] years; 1 247 480 women [41.9%]; mean [SD] age, 48.5 [9.9] years). At baseline, the prevalence of current smoking was significantly higher among men (1 069 470 men [61.9%]) than among women (20 962 women [1.7%]). Among current smokers at baseline, 649 732 men (60.8%) and 18 258 women (87.1%) attempted to quit smoking between 2004 and 2017.

The proportions of time-updated smoking status among men and women were as follows: continuous smokers, 419 738 men (24.3%) and 2704 women (0.2%); relapsed quitters, 191 940 men (11.1%) and 3834 women (0.3%); transient quitters, 92 587 men (5.4%) and 3619 women (0.3%); complete quitters, 365 205 men (21.1%) and 10 805 women (0.9%); and never smokers, 657 870 men (38.1%) and 1 226 518 women (98.3%). The prevalence rates of chronic viral hepatitis and liver cirrhosis were 7.5% (129 717 men) and 0.1% (1258 men), respectively, among men and 7.2% (89 690 women) and 0.01% (146 women), respectively, among women (data not shown). The detailed sociodemographic characteristics and lifestyle behaviors of the study participants are presented in Table 1.

**Table 1.** General Characteristics of Study Participants by Sex and Time-Updated Smoking Status Defined by Change of Smoking Behavior, 2002-2017^a^

Characteristic	Participants, No. (%)									
	Men (n = 1 727 340)					Women (n = 1 247 480)				
	Continuous smokers	Relapsed quitters	Transient quitters	Complete quitters	Never smokers	Continuous smokers	Relapsed quitters	Transient quitters	Complete quitters	Never smokers
Total No.	419 738	191 940	92 587	365 205	657 870	2704	3834	3619	10 805	1 226 518
Time since quitting, mean (SD), y	NA	3.2 (1.8)	11.8 (2.2)	13.0 (3.7)	NA	NA	3.9 (2.2)	12.1 (2.2)	14.6 (3.4)	NA
Age, mean (SD), y	39.5 (8.0)	40.7 (8.9)	43.2 (9.9)	43.1 (9.5)	46.0 (10.8)	48.2 (9.6)	50.5 (10.1)	51.2 (10.3)	49.7 (1.0)	48.5 (9.9)
Age range, y										
30-39	236 782 (56.3)	99 102 (51.6)	37 770 (40.8)	147 284 (40.3)	209 383 (31.8)	384 (14.2)	405 (10.5)	345 (9.5)	1287 (11.9)	209 205 (17.1)
40-49	133 557 (31.8)	61 457 (32.0)	31 208 (33.7)	129 048 (35.4)	211 289 (32.1)	1199 (44.3)	1491 (38.9)	1395 (38.6)	4528 (41.9)	473 995 (38.7)
50-59	39 239 (9.4)	23 025 (12.0)	16 232 (17.5)	64 064 (17.5)	144 613 (22.0)	723 (26.8)	1091 (28.5)	987 (27.3)	2897 (26.8)	342 718 (27.9)
60-69	9473 (2.3)	7635 (4.0)	6664 (7.2)	22 434 (6.1)	80 273 (12.2)	338 (12.5)	720 (18.8)	731 (20.2)	1755 (16.2)	178 633 (14.6)
≥70	687 (0.2)	721 (0.4)	713 (0.8)	2375 (0.7)	12 312 (1.9)	60 (2.2)	127 (3.3)	161 (4.5)	338 (3.1)	21 967 (1.8)
Income level										
Medical aid or first quartile	45 184 (10.8)	17 832 (9.3)	8290 (9.0)	35 832 (9.8)	63 917 (9.7)	917 (33.9)	1193 (31.1)	1015 (28.1)	3352 (31.0)	313 137 (25.5)
Second quartile	101 756 (24.2)	40 118 (20.9)	16 975 (18.3)	70 094 (19.2)	117 618 (17.9)	755 (27.9)	1099 (28.7)	1009 (27.9)	2964 (27.4)	239 458 (19.52)
Third quartile	150 629 (35.9)	67 625 (35.2)	30 493 (32.9)	118 510 (32.4)	201 315 (30.6)	569 (21.1)	828 (21.6)	856 (23.7)	2272 (21.0)	292 972 (23.9)
Fourth quartile	122 169 (29.1)	66 365 (34.6)	36 829 (39.8)	140 769 (38.6)	275 020 (41.8)	463 (17.1)	714 (18.6)	739 (20.4)	2217 (20.5)	380 951 (31.1)
Alcohol consumption										
Nondrinkers	79 937 (19.0)	37 763 (19.7)	18 532 (20.0)	73 827 (20.2)	286 309 (43.5)	1075 (39.8)	1805 (47.1)	1764 (48.7)	5353 (49.5)	995 649 (81.2)
<24 g/d	274 074 (65.3)	126 273 (65.8)	61 564 (66.5)	241 062 (66.0)	324 913 (49.4)	1347 (49.8)	1762 (45.9)	1656 (45.8)	4716 (43.7)	225 765 (18.4)
≥24 g/d	65 727 (15.7)	27 904 (14.5)	12 491 (13.5)	50 316 (13.8)	46 648 (7.1)	282 (10.4)	267 (7.0)	199 (5.5)	736 (6.8)	5104 (0.4)
Body mass index, mean (SD)^b^	23.7 (3.0)	23.9 (2.9)	24.0 (2.8)	23.9 (2.8)	24.1 (2.8)	23.4 (3.3)	23.4 (3.3)	23.5 (3.2)	23.4 (3.2)	23.5 (3.0)
<23	172 461 (41.1)	72 920 (38.0)	33 943 (36.7)	137 729 (37.7)	223 405 (34.0)	1305 (48.3)	1819 (47.4)	1700 (47.0)	5112 (47.3)	558 903 (45.6)
23 to <25	109 697 (26.1)	51 557 (26.9)	26 218 (28.3)	102 963 (28.2)	194 174 (29.5)	569 (21.0)	909 (23.7)	853 (23.6)	2543 (23.5)	306 677 (25.0)
≥25	137 580 (32.8)	67 463 (35.1)	32 426 (35.0)	124 513 (34.1)	240 291 (36.5)	830 (30.7)	1106 (28.9)	1066 (29.5)	3150 (29.2)	360 938 (29.4)
Physical activity										
No	201 684 (48.0)	92 095 (48.0)	43 211 (46.7)	176 670 (48.4)	293 467 (44.6)	1884 (69.7)	2681 (69.9)	2457 (67.9)	6991 (64.7)	816 547 (66.6)
1-2 d/wk	156 448 (37.3)	69 080 (36.0)	32 978 (35.6)	127 823 (35.0)	220 535 (33.5)	441 (16.3)	576 (15.0)	581 (16.1)	1995 (18.5)	215 988 (17.6)
3-4 d/wk	40 233 (9.6)	19 938 (10.4)	10 232 (11.0)	38 246 (10.5)	82 848 (12.6)	191 (7.0)	262 (6.8)	251 (6.9)	872 (8.1)	96 661 (7.9)
5-6 d/wk	8185 (2.0)	4001 (2.1)	2123 (2.3)	7834 (2.1)	19 979 (3.0)	56 (2.1)	86 (2.3)	91 (2.5)	274 (2.5)	27 891 (2.3)
7 d/wk	13 188 (3.1)	6826 (3.5)	4043 (4.4)	14 632 (4.0)	41 041 (6.3)	132 (4.9)	229 (6.0)	239 (6.6)	673 (6.2)	69 431 (5.7)
Pack-years										
<10	96 985 (23.1)	54 983 (28.6)	33 751 (36.5)	121 892 (33.4)	NA	1571 (58.0)	2569 (67.1)	2663 (73.5)	7144 (66.1)	NA
10 to <20	193 460 (46.1)	79 444 (41.4)	32 046 (34.6)	135 649 (37.1)	NA	672 (24.8)	709 (18.5)	524 (14.5)	1517 (14.0)	NA
≥20	129 293 (30.8)	57 513 (30.0)	26 790 (28.9)	107 664 (29.5)	NA	461 (17.1)	556 (14.5)	432 (11.9)	2144 (19.8)	NA

Abbreviation: NA, not applicable.

^a^ Information was collected from baseline (2002-2003).

^b^ Body mass index is calculated as weight in kilograms divided by height in meters squared.

### Risk of Cancer Incidence by Time-Updated Smoking Status and Years Since Quitting

During the mean (SD) follow-up period of 13.4 (0.1) years, 196 829 incident cases of primary cancer were ascertained, with 117 805 cases (6.82%) in men and 73 946 cases (5.93%) in women. The cancer incidence rates among participants included in this study were lower compared with those among individuals excluded from this study (11.82% for men and 11.76% for women) (eTable 2 in Supplement 1). In multivariable-adjusted models, complete quitters demonstrated a significantly lower risk of cancer compared with continuous smokers among men (all sites aHR, 0.83 [95% CI, 0.80-0.86]; lung aHR, 0.58 [95% CI, 0.53-0.62]; liver aHR, 0.73 [95% CI, 0.64-0.82]; stomach aHR, 0.86 [95% CI, 0.79-0.93]; colorectum aHR, 0.80 [95% CI, 0.72-0.89]). There was a linear decreasing trend in the cancer risk in the order of continuous smokers, relapsed quitters, transient quitters, complete quitters, and never smokers (*P* for trend < .001) (Table 2).

**Table 2.** HRs of Cancer Incidence by Time-Updated Smoking Status Among Men

Cancer site and time-updated smoking status	Participants, No.	Incident cancer cases, No.	Person-years, No.	Unadjusted HR (95% CI)	*P* value	Age-adjusted HR (95% CI)	*P* value	Multivariable-adjusted HR (95% CI)^a^	*P* value
All sites (except melanoma)									
Continuous smokers	419 738	25 677	5 600 238	1 [Reference]	NA	1 [Reference]	NA	1 [Reference]	NA
Relapsed quitters	191 940	12 650	2 559 519	1.08 (1.04-1.12)	<.001	0.93 (0.89-0.97)	<.001	0.94 (0.90-0.98)	.004
Transient quitters	92 587	7101	1 233 624	1.26 (1.20-1.32)	<.001	0.84 (0.80-0.89)	<.001	0.87 (0.83-0.92)	<.001
Complete quitters	365 205	26 100	4 868 252	1.17 (1.13-1.21)	<.001	0.81 (0.78-0.83)	<.001	0.83 (0.80-0.86)	<.001
Never smokers	657 870	46 277	8 765 082	1.15 (1.12-1.19)	<.001	0.61 (0.59-0.62)	<.001	0.57 (0.55-0.59)	<.001
* P* for trend	NA	NA	NA	<.001	NA	<.001	NA	<.001	NA
Lung									
Continuous smokers	419 738	4994	5 600 238	1 [Reference]	NA	1 [Reference]	NA	1 [Reference]	NA
Relapsed quitters	191 940	2560	2 559 519	1.12 (1.03-1.23)	.01	0.86 (0.79-0.94)	<.001	0.90 (0.82-0.98)	.02
Transient quitters	92 587	1349	1 233 624	1.23 (1.10-1.37)	<.001	0.64 (0.57-0.72)	<.001	0.72 (0.64-0.81)	<.001
Complete quitters	365 205	4075	4 868 252	0.94 (0.87-1.02)	.11	0.52 (0.48-0.56)	<.001	0.58 (0.53-0.62)	<.001
Never smokers	657 870	3950	8 765 082	0.51 (0.47-0.55)	<.001	0.18 (0.17-0.20)	<.001	0.16 (0.14-0.17)	<.001
* P* for trend	NA	NA	NA	<.001	NA	<.001	NA	<.001	NA
Liver									
Continuous smokers	419 738	2423	5 600 238	1 [Reference]	NA	1 [Reference]	NA	1 [Reference]	NA
Relapsed quitters	191 940	1099	2 559 519	0.99 (0.86-1.15)	.93	0.87 (0.75-1.00)	.06	0.87 (0.75-1.01)	.07
Transient quitters	92 587	598	1 233 624	1.12 (0.94-1.34)	.20	0.78 (0.65-0.94)	.008	0.79 (0.65-0.95)	.01
Complete quitters	365 205	2175	4 868 252	1.03 (0.92-1.16)	.57	0.74 (0.66-0.83)	<.001	0.73 (0.64-0.82)	<.001
Never smokers	657 870	3551	8 765 082	0.94 (0.85-1.04)	.23	0.52 (0.47-0.58)	<.001	0.58 (0.51-0.66)	<.001
* P* for trend	NA	NA	NA	.28	NA	<.001	NA	<.001	NA
Stomach									
Continuous smokers	419 738	5495	5 600 238	1 [Reference]	NA	1 [Reference]	NA	1 [Reference]	NA
Relapsed quitters	191 940	2323	2 559 519	0.93 (0.84-1.02)	.13	0.82 (0.74-0.91)	<.001	0.84 (0.76-0.93)	<.001
Transient quitters	92 587	1348	1 233 624	1.12 (0.99-1.26)	.08	0.80 (0.71-0.91)	<.001	0.83 (0.73-0.94)	.004
Complete quitters	365 205	5423	4 868 252	1.14 (1.05-1.23)	.001	0.83 (0.77-0.90)	<.001	0.86 (0.79-0.93)	<.001
Never smokers	657 870	8039	8 765 082	0.94 (0.87-1.01)	.07	0.55 (0.51-0.60)	<.001	0.55 (0.50-0.60)	<.001
* P* for trend	NA	NA	NA	.60	NA	<.001	NA	<.001	NA
Colon and rectum									
Continuous smokers	419 738	3552	5 600 238	1 [Reference]	NA	1 [Reference]	NA	1 [Reference]	NA
Relapsed quitters	191 940	1604	2 559 519	0.99 (0.87-1.12)	.86	0.87 (0.76-0.98)	.03	0.90 (0.79-1.03)	.12
Transient quitters	92 587	879	1 233 624	1.13 (0.96-1.32)	.14	0.79 (0.67-0.92)	.003	0.85 (0.72-0.99)	.05
Complete quitters	365 205	3294	4 868 252	1.07 (0.97-1.18)	.20	0.76 (0.69-0.85)	<.001	0.80 (0.72-0.89)	<.001
Never smokers	657 870	5872	8 765 082	1.06 (0.97-1.16)	.21	0.59 (0.54-0.65)	<.001	0.70 (0.63-0.79)	<.001
* P* for trend	NA	NA	NA	.13	NA	<.001	NA	<.001	NA

Abbreviations: HR, hazard ratio; NA, not applicable.

^a^ HRs and 95% CIs were adjusted for age (in year, continuous and quadratic terms), body mass index (in continuous term), income level, physical activity, alcohol consumption, and pack-years. HRs for liver cancer were further adjusted for chronic viral hepatitis and liver cirrhosis.

Among women, quitting smoking was not significantly associated with a reduced risk of all-site, lung, and colorectal cancers (eTable 3 in Supplement 1). The risk of liver and stomach cancers among women was not presented because of the lack of cancer cases among women.

**Figure 1.**
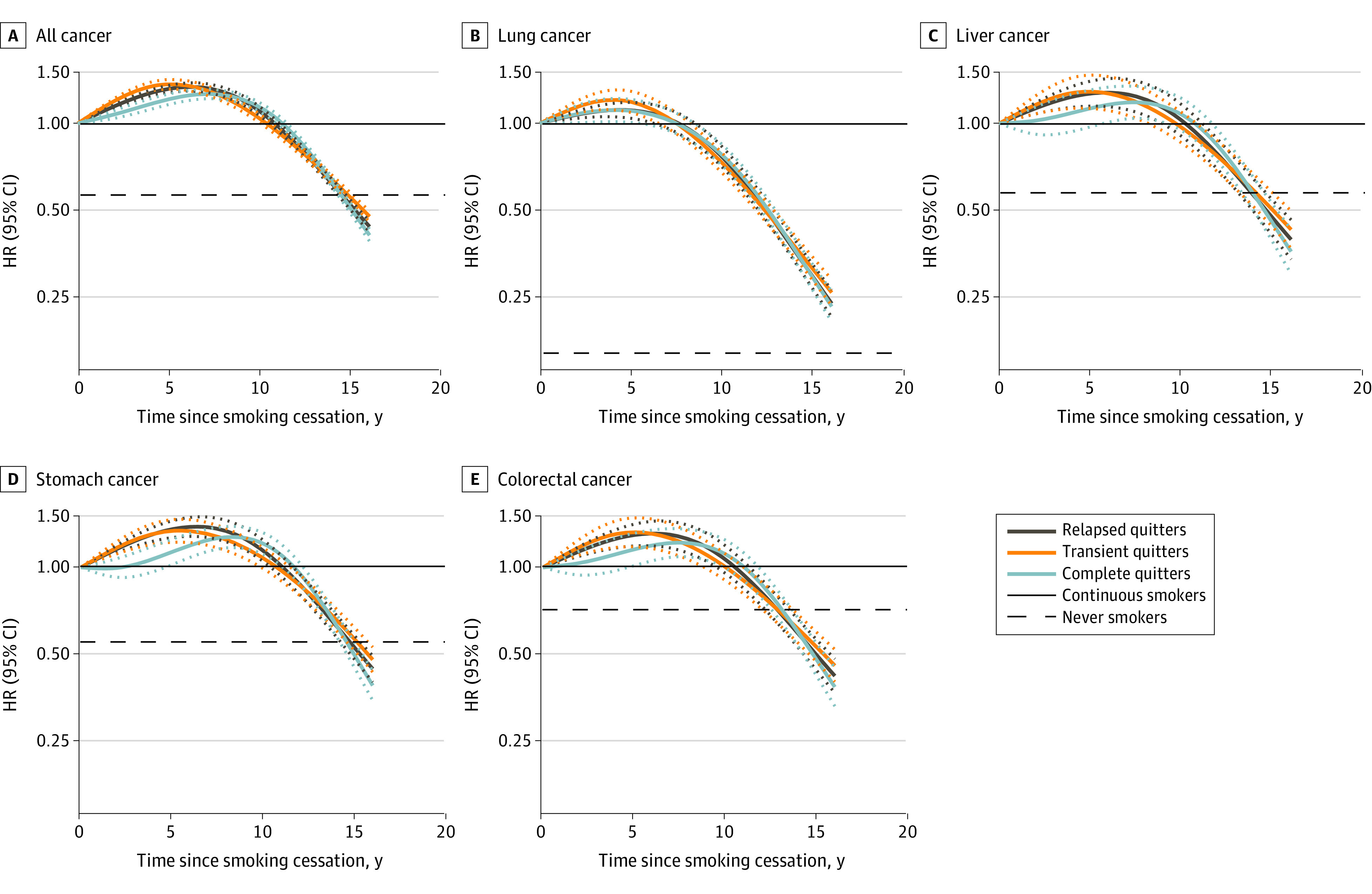
Association of Duration of Smoking Cessation With Cancer Incidence Among Men Hazard ratios (HRs) and 95% CIs were adjusted for age (in year, continuous and quadratic terms), body mass index (in continuous term), income level, physical activity, alcohol consumption, and pack-years. HRs for liver cancer were further adjusted for chronic viral hepatitis and liver cirrhosis. Dotted lines represent 95% CIs. Splines have 4 knots at 5th, 35th, 65th, and 95th percentiles of years since quitting.

In the restricted cubic spline analyses, a minor disparity in cancer incidence was observed at the initiation of quitting among subgroups of quitters, but no substantial divergence was noted in the time course of cancer risk. The lung cancer risk slightly increased until 5 years after quitting and gradually decreased thereafter, reaching 50% of that of continuous smokers after 12 years. For all-site cancer and other cancer types (ie, cancer of the liver, stomach, and colorectum), the risks peaked at approximately 7 years after quitting and gradually decreased thereafter, reaching 50% of that of continuous smokers after 15 years (Figure 1). In multivariable-adjusted Cox proportional hazards regression models, smoking cessation was associated with a reduced risk of all-site cancer after 10 years of quitting smoking (aHR for quitting <10 years, 1.12 [95% CI, 1.08-1.16]; aHR for quitting 10 to <15 years, 0.76 [95% CI, 0.73-0.79]; aHR for quitting ≥15 years, 0.41 [95% CI, 0.38-0.44]). This risk reduction in all-site cancer was generally consistent with that in the lung, liver, stomach, and colorectal cancers, particularly with significant magnitude of reduction in the risk of lung cancer (Table 3).

**Table 3.** Multivariable-Adjusted HRs of Incident Cancer by Years Since Quitting Among Men

Cancer site and time since quitting	Participants, No.	Incident cancer cases, No.	Person-years, No.	Multivariable-adjusted HR (95% CI)^a^	*P* value
All sites (except melanoma)					
Continuous smokers	419 738	25 677	5 600 238	1 [Reference]	NA
<10 y	311 319	26 615	4 145 611	1.12 (1.08-1.16)	<.001
10 to <15 y	224 147	14 906	2 987 971	0.76 (0.73-0.79)	<.001
≥15 y	114 266	4330	1 527 813	0.41 (0.38-0.44)	<.001
Never smokers	657 870	46 277	8 765 082	0.57 (0.55-0.59)	<.001
*P* for trend	NA	NA	NA	<.001	NA
Lung					
Continuous smokers	419 738	4994	5 600 238	1 [Reference]	NA
<10 y	311 319	5147	4 145 611	0.98 (0.91-1.06)	.66
10 to <15 y	224 147	2310	2 987 971	0.53 (0.48-0.58)	<.001
≥15 y	114 266	527	1 527 813	0.23 (0.19-0.27)	<.001
Never smokers	657 870	3950	8 765 082	0.16 (0.14-0.17)	<.001
*P* for trend	NA	NA	NA	<.001	NA
Liver					
Continuous smokers	419 738	2423	5 600 238	1 [Reference]	NA
<10 y	311 319	2283	4 145 611	1.03 (0.91-1.16)	.63
10 to <15 y	224 147	1312	2 987 971	0.70 (0.61-0.81)	<.001
≥15 y	114 266	277	1 527 813	0.27 (0.21-0.35)	<.001
Never smokers	657 870	3551	8 765 082	0.58 (0.51-0.67)	<.001
*P* for trend	NA	NA	NA	<.001	NA
Stomach					
Continuous smokers	419 738	5495	5 600 238	1 [Reference]	NA
<10 y	311 319	5249	4 145 611	1.08 (1.00-1.17)	.06
10 to <15 y	224 147	3003	2 987 971	0.77 (0.70-0.85)	<.001
≥15 y	114 266	842	1 527 813	0.40 (0.34-0.47)	<.001
Never smokers	657 870	8039	8 765 082	0.55 (0.51-0.60)	<.001
*P* for trend	NA	NA	NA	<.001	NA
Colon and rectum					
Continuous smokers	419 738	3552	5 600 238	1 [Reference]	NA
<10 y	311 319	3421	4 145 611	1.10 (0.99-1.22)	.07
10 to <15 y	224 147	1785	2 987 971	0.71 (0.62-0.80)	<.001
≥15 y	114 266	571	1 527 813	0.41 (0.34-0.50)	<.001
Never smokers	657 870	5872	8 765 082	0.71 (0.63-0.80)	<.001
*P* for trend	NA	NA	NA	<.001	NA

Abbreviations: HR, hazard ratio; NA, not applicable.

^a^ HRs and 95% CIs were adjusted for age (in year, continuous and quadratic terms), body mass index (in continuous term), income level, physical activity, alcohol consumption, and pack-years. HRs for liver cancer were further adjusted for chronic viral hepatitis and liver cirrhosis.

### Risk of Cancer Incidence by Age at Smoking Cessation

**Figure 2.**
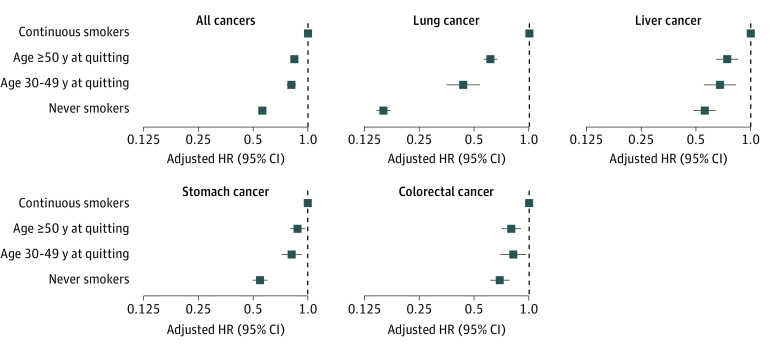
Multivariable-Adjusted Hazard Ratios (HRs) of Incident Cancer by Age at Quitting Among Men HRs and 95% CIs were adjusted for age (in year, continuous and quadratic terms), body mass index (in continuous term), income level, physical activity, alcohol consumption, and pack-years. HRs for liver cancer were further adjusted for chronic viral hepatitis and liver cirrhosis.

We observed that, compared with continuous smokers, quitting smoking at any age was associated with a reduced risk of cancers. Compared with continuous smokers, the aHRs of those who quit smoking after the age of 50 were 0.84 (95% CI, 0.81-0.88) for all sites, 0.61 (95% CI, 0.56-0.66) for lungs, 0.74 (95% CI, 0.64-0.85) for liver, 0.88 (95% CI, 0.80-0.96) for stomach, and 0.80 (95% CI, 0.71-0.90) for colorectum. The aHRs of those who quit smoking before the age of 50 years were 0.81 (95% CI, 0.77-0.86) for all sites, 0.43 (95% CI, 0.35-0.53) for lung, 0.68 (95% CI, 0.55-0.83) for liver, 0.82 (95% CI, 0.72-0.93) for stomach, and 0.82 (95% CI, 0.69-0.97) for colorectum (Figure 2). We found a significant difference in the reduction of lung cancer risk between those who quit before the age of 50 vs and those who quit after the age of 50 years; however, the differences in the risk reduction of other cancer types were not statistically significant.

### Sensitivity Analysis

General characteristics of the study participants in the sensitivity analyses are presented in eTable 4 in Supplement 1. In the sensitivity analyses using the self-reported smoking status, the results were generally consistent with those of the primary analyses using the time-updated smoking status (eTables 5 and 6 in Supplement 1). However, the estimates obtained from the sensitivity analyses showed a linear decrease in the risk with increasing years since quitting, without an increase in the risk after quitting, contrary to those reported in the primary analyses (eTable 7, eFigure 7, and eFigure 8 in Supplement 1).

## Discussion

In this large population-based cohort study, we found that, compared with continuous smokers, smoking cessation among complete or transient quitters was associated with a lower risk of all-site, lung, liver, stomach, and colorectal cancers, reaffirming a linear decreasing trend in the order of continuous smokers, relapsed quitters, transient quitters, complete quitters, and never smokers. A larger relative decrease in lung cancer risk was observed among complete quitters. Furthermore, the results demonstrated that, after smoking cessation, the cancer risk showed a slightly higher value for approximately a decade and then decreased linearly over time, reaching 50% of the risk associated with continued smoking at 15 years or more after quitting. Lung cancer risk decreased 3 years earlier than that of other cancer types, accompanied by a larger relative reduction. These findings indicate that sustained long-term smoking cessation, even if it includes a period of relapse, can substantially reduce the cancer risk associated with continued smoking.

These findings are consistent with those reported in previous studies^8,27,28,29,30,31,32^ that evaluated the time course of cancer risk after smoking cessation. A meta-analysis^33^ conducted by the International Agency for Research on Cancer in 2007 reported that lung cancer risk decreased to half that of continuous smokers at 10 to 15 years after smoking cessation. Furthermore, several cohorts with stomach and colorectal cancers showed that past smokers who had sustained smoking cessation over a longer duration (ranging from ≥10 to ≥20 years for stomach cancer and ≥10 to ≥40 years for colorectal cancer) exhibited a reduced risk of cancer compared with those who had quit smoking for a shorter period.^7,34^ A case-control study^35^ reported that the risk of liver cancer among past smokers who had quit for 10 years or more did not significantly differ from that among never smokers.

In the present study, we observed an increase in the cancer risk for approximately 7 years following smoking cessation. The observed increase in the cancer risk following smoking cessation may be attributed to the inclusion of individuals who had already accumulated substantial damage caused by smoking, known as *sick quitters*. The possibility of including sick quitters in our study arose because the participants initiated their quitting attempts since baseline. This explanation is supported by the findings of our sensitivity analysis, which demonstrated a subsequent decrease in the cancer risk without any initial increase after cessation, which contrasts with that reported in the primary analysis. In sensitivity analysis, the inclusion of sick quitters almost was not possible because we included quitters (past smokers) who had already stopped smoking before baseline. This trend aligns with the well-documented phenomenon of escalated medical use and associated expenses among those who have just quit.^36,37^

Moreover, the analyses in this study provided evidence regarding the impact of age at smoking cessation on the reduction in cancer risk. This study indicates that smoking cessation at any age is associated with a decreased risk of developing cancer. Specifically, individuals who quit smoking before the age of 50 years had a greater reduction in lung cancer risk than those who quit smoking after the age of 50 years. This finding aligns with that of a previous case-control study^38^ in the United Kingdom, which highlighted that smoking cessation before middle age mitigates most of the subsequent risk of lung cancer. However, for liver, stomach, and colorectal cancers, we did not observe significant differences between quitting smoking before and after the age of 50 years, which is consistent with that reported in a meta-analysis.^7^ This may be attributed to the long-lasting effects of smoking on these cancer types.^3,39^ In addition, considering that several studies on mortality have shown a substantial reduction in the excess risk associated with continued smoking,^18,19,27,40,41^ we suggest that a longer period of smoking cessation is necessary to fully evaluate the outcomes according to the age at which individuals quit smoking.

Our findings were strengthened by the large population-based cohort design with a complete follow-up rate through linkages to the national cancer registry data. To ensure accurate determination of the time-updated smoking status, we used long-term repeated measurements of smoking behaviors. This rigorous approach minimized the potential for misclassification when identifying the time-updated smoking status. Furthermore, the study adopted an exclusion criterion for individuals who were past smokers at baseline, and the years of smoking cessation were calculated for those who started to quit smoking during the study period. This method helps alleviate the confounding effect of age on the association of the duration of smoking cessation with cancer risk. Considering that older individuals who quit smoking are more likely to have a longer duration of smoking cessation, it is important to address this confounding effect, particularly in studies involving participants spanning various age ranges. Moreover, the findings from this study involving the Korean population could substantively contribute to the existing body of knowledge, given that the lack of sufficient evidence regarding the association between time-updated smoking status, duration of smoking cessation, and the consequent reduction in cancer risk except for lung cancer, particularly within the Asia-Pacific region where smoking rates remain high.

### Limitations

This study has limitations. First, the study relied on the consecutive examinations of biennial health screens to define the time-updated smoking status, which introduces the possibility of a selection bias, despite the high participation rate of the NHIS health examination (eg, 74% in 2019).^42^ The incidence rate of cancer among the participants in our study (6.82% in men and 5.93% in women) was significantly lower than that observed in the excluded participants (11.82% in men and 11.76% in women). Our study population consisted of individuals in good health, characterized by a relatively younger age demographic, a lower prevalence of tobacco use, and a higher economic status (eTable 2 in Supplement 1). Second, the follow-up period was insufficient to identify whether there was a difference in cancer risk reduction according to the age at smoking cessation. Third, owing to the unavailability of the exact date of smoking cessation or relapse, the duration of smoking cessation was estimated by assuming that a quit date is the starting point of the cycle. Fourth, there exists the possibility that a competing risk either hinders the observation of cancer incidence or modifies the chance that cancer incidence occurs. A competing risk, such as noncancerous death, was observed to be more frequent among male never smokers compared with smokers, introducing hindrance of the observation of cancer. Fifth, it is important to pay attention to the interpretation of the analysis results for women because of the lack of statistical power of the analysis resulting from the limited number of female smokers.

## Conclusions

The findings of this cohort study suggest that sustained smoking cessation is associated with delayed or prevented cancer onset, particularly after 10 years or more since smoking cessation. Cancer risk was reduced by approximately 50% at 15 years or more since quitting compared with that associated with continued smoking. In particular, the time course of lung cancer risk after smoking cessation proceeded 3 years earlier than that of other cancer types. Furthermore, smoking cessation at any age helps reduce the cancer risk, and early cessation before middle age, especially for lung cancer, exhibited a substantial risk reduction. Our findings emphasize the significance of promoting smoking cessation, offering appropriate support and resources for sustained cessation, and encouraging cessation at an early age to reduce the risk of cancer.

## Notes

Supplement 1. **eAppendix.** Supplemental Methods **eTable 1.** The Demographic Characteristics in Participants With Complete Value of Smoking Status Every Two Years and Participants With Imputed Smoking Status in This Study **eTable 2.** General Characteristics of Study Participants and the Excluded Participants by Gender in 2002 and 2003 **eTable 3.** Hazard Ratios of Cancer Incidence by Time-Updated Smoking Status Among Women **eTable 4.** General Characteristics of Study Participants Who Underwent Health Screening From 2006 to 2008 **eTable 5.** Hazard Ratios of Cancer Incidence Among Male Participants Who Underwent Health Screening From 2006 to 2008 by Self-Reported Smoking Status **eTable 6.** Hazard Ratios of Cancer Incidence Among Female Participants Who Underwent Health Screening From 2006 to 2008 by Self-Reported Smoking Status **eTable 7.** Hazard Ratios of Cancer Incidence Among Male Participants Who Underwent Health Screening From 2006 to 2008 by Categorized Years Since Quitting **eFigure 1.** Study Population Flowchart **eFigure 2.** Definition of Time-Updated Smoking Status and Duration of Smoking Cessation During Follow-Up (2009-2019) **eFigure 3.** Cancer Incidence Rates per 100,000 Persons in Male Participants at Each Time Point From 2006 to 2019 **eFigure 4.** Cancer Incidence Rates per 100,000 Persons in Female Participants at Each Time Point From 2006 to 2019 **eFigure 5.** Study Population Flowchart for Sensitivity Analysis **eFigure 6.** Definition of Smoking Status and Duration of Smoking Cessation in the Sensitivity Analysis **eFigure 7.** Hazard Ratio and 95% Confidence Intervals of Self-Reported Past Smokers Compared to Current Smokers According to Years Since Quitting Among Men Who Underwent Health Screening From 2006 to 2008 **eFigure 8.** Hazard Ratio and 95% Confidence Intervals of Self-Reported Past Smokers Compared to Current Smokers According to Years Since Quitting Among Women Who Underwent Health Screening From 2006 to 2008

Supplement 2.
Data Sharing Statement
