# Supplementary material for: Cancer Risk Following Smoking Cessation in Korea
Source: JAMA Netw Open. 2024 Feb 6;7(2):e2354958. doi: 10.1001/jamanetworkopen.2023.54958 (PMC10848071; doi:10.1001/jamanetworkopen.2023.54958)
Supplement: Supplement 1. — eAppendix. Supplemental Methods eTable 1. The Demographic Characteristics in Participants With Complete Value of Smoking Status Every Two Years and Participants With Imputed Smoking Status in This Study eTable 2. General Characteristics of Study Participants and the Excluded Participants by Gender in 2002 and 2003 eTable 3. Hazard Ratios of Cancer Incidence by Time-Updated Smoking Status Among Women eTable 4. General Characteristics of Study Participants Who Underwent Health Screening From 2006 to 2008 eTable 5. Hazard Ratios of Cancer Incidence Among Male Participants Who Underwent Health Screening From 2006 to 2008 by Self-Reported Smoking Status eTable 6. Hazard Ratios of Cancer Incidence Among Female Participants Who Underwent Health Screening From 2006 to 2008 by Self-Reported Smoking Status eTable 7. Hazard Ratios of Cancer Incidence Among Male Participants Who Underwent Health Screening From 2006 to 2008 by Categorized Years Since Quitting eFigure 1. Study Population Flowchart eFigure 2. Definition of Time-Updated Smoking Status and Duration of Smoking Cessation During Follow-Up (2009-2019) eFigure 3. Cancer Incidence Rates per 100,000 Persons in Male Participants at Each Time Point From 2006 to 2019 eFigure 4. Cancer Incidence Rates per 100,000 Persons in Female Participants at Each Time Point From 2006 to 2019 eFigure 5. Study Population Flowchart for Sensitivity Analysis eFigure 6. Definition of Smoking Status and Duration of Smoking Cessation in the Sensitivity Analysis eFigure 7. Hazard Ratio and 95% Confidence Intervals of Self-Reported Past Smokers Compared to Current Smokers According to Years Since Quitting Among Men Who Underwent Health Screening From 2006 to 2008 eFigure 8. Hazard Ratio and 95% Confidence Intervals of Self-Reported Past Smokers Compared to Current Smokers According to Years Since Quitting Among Women Who Underwent Health Screening From 2006 to 2008 [file jamanetwopen-e2354958-s001.pdf]

Supplemental Online Content

Park E, Kang HY, Lim MK, Kim B, Oh JK. Cancer risk following smoking cessation in Korea. *JAMA Netw Open*. 2024;7(2):e2354958. doi:10.1001/jamanetworkopen.2023.54958

eAppendix. Supplemental Methods

eTable 1. The Demographic Characteristics in Participants With Complete Value of Smoking Status Every Two Years and Participants With Imputed Smoking Status in This Study

eTable 2. General Characteristics of Study Participants and the Excluded Participants by Gender in 2002 and 2003

eTable 3. Hazard Ratios of Cancer Incidence by Time-Updated Smoking Status Among Women

eTable 4. General Characteristics of Study Participants Who Underwent Health Screening From 2006 to 2008

eTable 5. Hazard Ratios of Cancer Incidence Among Male Participants Who Underwent Health Screening From 2006 to 2008 by Self-Reported Smoking Status

eTable 6. Hazard Ratios of Cancer Incidence Among Female Participants Who Underwent Health Screening From 2006 to 2008 by Self-Reported Smoking Status

eTable 7. Hazard Ratios of Cancer Incidence Among Male Participants Who Underwent Health Screening from 2006 to 2008 by Categorized Years Since Quitting

eFigure 1. Study Population Flowchart

eFigure 2. Definition of Time-Updated Smoking Status and Duration of Smoking Cessation During Follow-Up (2009-2019)

eFigure 3. Cancer Incidence Rates per 100,000 Persons in Male Participants at Each Time Point From 2006 to 2019

eFigure 4. Cancer Incidence Rates per 100,000 Persons in Female Participants at Each Time Point From 2006 to 2019

eFigure 5. Study Population Flowchart for Sensitivity Analysis

eFigure 6. Definition of Smoking Status and Duration of Smoking Cessation in the Sensitivity Analysis

eFigure 7. Hazard Ratio and 95% Confidence Intervals of Self-Reported Past Smokers Compared to Current Smokers According to Years Since Quitting Among Men Who Underwent Health Screening From 2006 to 2008

eFigure 8. Hazard Ratio and 95% Confidence Intervals of Self-Reported Past Smokers Compared to Current Smokers According to Years Since Quitting Among Women Who Underwent Health Screening From 2006 to 2008

This supplemental material has been provided by the authors to give readers additional information about their work.

© 2024 Park E et al. *JAMA Network Open*

eAppendix. Supplemental Methods

Study Participants

For the present analysis, 198,817 individuals who had been diagnosed with cancer or died before January 2006, that is, the initiation period for the observation of cancer incidence, were excluded from the study. Additionally, 893,219 participants reported being past smokers or who did not provide smoking information (i.e. smoking duration and/or daily amount of smoking) during the baseline period. Also, 3,317,961 participants who did not provide smoking information in two or more consecutive cycles or completed only the baseline questionnaire were excluded to ensure the accurate determination of the participants' time-updated smoking status. Furthermore, 152,065 participants without information on income level, body mass index (BMI), alcohol consumption, or physical activity, during the baseline were excluded. After exclusion, 2,974,820 participants were included in the analysis (eFigure 1).

Multiple assessments and missing data

For participants with multiple assessments of smoking status within a single cycle, we determined their smoking status by adopting the initial assessments of health screenings conducted during that cycle. In cases where smoking status was unavailable, we imputed missing data using assessments from the immediately preceding cycle (28.3% of participants). Therefore, two or more consecutive occurrences of missing values were not imputed, resulting in their exclusion from the study participants.

Covariates

Baseline covariates included age, sex, income level, BMI, alcohol consumption, physical activity, chronic viral hepatitis (for liver cancer), and liver cirrhosis (for liver cancer). Income level was determined using the national health insurance premiums 20-quantile, which was reclassified into quartiles: first (including medical aid), second, third, or fourth. BMI was calculated by dividing the weight in kilograms by the square of height in meres, measured using calibrated equipment by the staff of medical institutions. Self-reported alcohol consumption and physical activity levels were collected using structured questionnaires. Alcohol consumption was assessed by the daily amount of alcohol intake, calculated based on the type of liquor and quantity of drinks consumed. Alcohol consumption was categorized as non-drinkers, ≤24g daily, or >24g daily. Physical activity was categorized based on the frequency of activity per week: rarely, 1–2 days, 3–4 days, 5–6 days, or almost every day. Pack-years were calculated by multiplying the number of packs of cigarettes smoked per day by the number of years the person had smoked. Additionally, the presence of chronic viral hepatitis (ICD-10: B15-B19 or K703) and liver cirrhosis (ICD-10: K703) were ascertained.

Tests for Proportional Hazards Assumption

We tested that the proportional hazards assumption in all models, considering two main exposure variables: time-updated smoking status (continuous smokers, complete quitters, transient quitters, relapsed quitters, and never smokers) and smoking cessation categorized by duration (continuous smokers, all quitters in the quitting duration categories: <10 years, 10 to <15 years, ≥15 years, and never smokers). For each outcome variable, the models incorporated the cross-product terms between each exposure variable and log-transformed follow-up time. There was no evidence of violations of this assumption, as P values from the Wald tests for the cross-product terms were all greater than 0.05. Additionally, we reconfirmed the proportional hazards assumption by testing the correlation between the Schoenfeld residuals and follow-up time.

Sensitivity Analysis

Background

In this study, all quitters began their quitting attempt after baseline, which raised the possibility that smoking cessation might have been influenced by their illness. For checking possibility of sick quitter effect, we conducted sensitivity analysis using a subset of the parent data which is available of information on self-reported smoking cessation years before baseline. These analyses were performed in a manner similar to that of the primary analyses, using the variables of smoking status and duration of smoking cessation obtained through self-reported questionnaires.

Data source and Study Participants

For sensitivity analyses, we extracted 5,909,170 participants aged 30 years and above who underwent health screening in 2006 to 2008 from the health insurance claims data from the National Health Insurance Service. A total of 182,744 individuals who had been diagnosed with cancer or deceased prior to January 2009, the initiation of observation of cancer incidence, were excluded from the study cohort. Additionally, 234,331 participants who did not provide smoking information during the baseline period (2006 to 2008) were also excluded. Furthermore, 206,106 participants who did not provide information on variables such as income level, body mass index (BMI), alcohol consumption, and physical activity during the baseline period were excluded. After the exclusions, a total of 5,285,989 participants were included in the analyses.

Exposures

We defined smoking status using self-reported questionnaire in baseline (2006 to 2008). The study participants were classified into three categories like as current smokers, past smokers, and never smokers. The duration of smoking cessation was obtained from a self-reported questionnaire administered to past smokers. This allowed us to exclude individuals who quit smoking due to illness, unlikely in the primary analysis.

Statistical analysis

Sensitivity analyses process was conducted in a similar manner to the primary analyses. To identify the association between smoking cessation and study outcomes, we employed a Cox proportional hazards regression model with person-years as the time scale and restricted cubic spline model. Hazard ratios and 95% confidence intervals were adjusted for age (in year, continuous and quadratic terms), body-mass index (in continuous term), income level (medical aid/1st quartile, 2nd quartile, 3rd quartile, or 4th quartile), physical activity (no, 1-2 days per week, 3-4 days per week, 5-6 days per week, or 7 days per week), and alcohol consumption (non-drinkers, <24g per day, or ≥24g per day) excluding pack-year due to its unavailability unlike the primary analyses. Hazard ratios for liver cancer were further adjusted for chronic viral hepatitis (negative or positive) and liver cirrhosis (negative or positive).

© 2024 Park E et al. *JAMA Network Open*

eTable 1. The Demographic Characteristics in Participants With Complete Value of Smoking Status Every Two Years and Participants With Imputed Smoking Status in This Study

Characteristic	Men			Women		
	Study population	With complete value	With imputed value	Study population	With complete value	With imputed value
Total, n	1,727,340	1,335,374	391,966	1,247,480	796,535	450,945
Person-years	23,026,715	17,168,723	5,857,992	16,705,859	10,219,222	6,486,637
All cancer, n (%)	117,805	90,939 (6.81)	26,866 (6.85)	73,976	43,570 (5.47)	30,406 (6.74)
Lung cancer, n (%)	16,928	12,953 (0.97)	3,975 (1.01)	6,237	4,620 (0.58)	1,617 (0.36)
Liver cance, n (%)	9,846	7,745 (0.58)	2,101 (0.54)	2,620	1,593 (0.20)	1,027 (0.23)
Stomach cancer, n (%)	22,628	17,627 (1.32)	5,001 (1.28)	7,984	5,177 (0.65)	2,807 (0.62)
Colorectal cancer, n (%)	15,201	11,751 (0.88)	3,450 (0.88)	7,984	5,098 (0.64)	2,886 (0.64)
Age, n (%)						
30 to 39	730,321	543,179 (40.68)	187,142 (47.74)	211,626	152,639 (18.63)	58,987 (13.08)
40 to 49	566,559	458,027 (32.80)	108,532 (27.69)	482,608	305,729 (38.38)	176,879 (39.22)
50 to 59	287,173	214,183 (16.04)	72,990 (18.62)	348,416	218,720 (27.46)	129,696 (28.76)
60 to 69	126,479	107,310 (8.04)	19,169 (4.89)	182,177	100,972 (12.68)	81,205 (18.01)
70 or more	16,808	12,675 (2.45)	4,133 (1.05)	22,653	18,475 (2.85)	4,178 (0.93)
Family income, n (%)						
Medical aid or 1st quartile	171,055	132,194 (9.90)	38,861 (9.91)	319,614	211,662 (26.57)	107,952 (23.94)
2nd quartile	346,561	260,458 (19.50)	86,103 (21.97)	245,285	153,301 (19.25)	91,984 (20.40)
3rd quartile	568,572	436,969 (32.72)	131,603 (33.58)	297,497	184,553 (23.17)	112,944 (25.05)
4th quartile	641,152	505,753 (37.87)	135,399 (34.54)	385,084	247,019 (31.01)	138,065 (30.62)
Pack-years, n (%)						
Never smokers	657,870	503,602 (37.71)	154,268 (39.36)	1,226,518	783,765 (98.40)	442,753 (98.18)
< 10	307,611	237,747 (17.80)	69,864 (17.82)	13,947	8,387 (1.05)	5,560 (1.23)
19-10	440,599	338,078 (25.32)	102,521 (26.16)	3,422	1,958 (0.25)	1,464 (0.32)
>=20	246,174	192,039 (14.38)	54,135 (13.81)	1,853	1,217 (0.15)	636 (0.14)
missing	75,086	63,908 (4.79)	11,178 (2.85)	1,740	1,208 (0.15)	532 (0.12)
Alcohol consumption, n (%)						
Non	496,368	390,232 (29.22)	106,136 (27.08)	1,005,646	644,137 (80.87)	361,509 (80.17)
< 24g per day	1,027,886	790,743 (59.22)	237,143 (60.50)	235,246	148,782 (18.68)	86,464 (19.17)
>= 24g per day	203,086	154,399 (11.56)	48,687 (12.42)	6,588	3,616 (0.45)	2,972 (0.66)
BMI, n (%)						
<23 kg/m2	640,458	509,990 (38.19)	130,468 (33.29)	568,839	373,739 (46.92)	195,100 (43.26)
23-24.9	484,609	373,030 (27.93)	111,579 (28.47)	311,551	196,956 (24.73)	114,595 (25.41)
>=25	602,273	452,354 (33.87)	149,919 (38.25)	367,090	225,840 (28.35)	141,250 (31.32)
Physical activity, n (%)						
No	807,127	612,930 (45.90)	194,197 (49.54)	830,560	528,569 (66.36)	301,991 (66.97)
1-2 days per week	606,864	473,011 (35.42)	133,853 (34.15)	219,581	143,082 (17.96)	76,499 (16.96)
3-4 days per week	191,497	151,068 (11.31)	40,429 (10.31)	98,237	63,408 (7.96)	34,829 (7.72)
5-6 days per week	42,122	33,204 (2.49)	8,918 (2.28)	28,398	18,022 (2.26)	10,376 (2.30)
7 days per week	79,730	65,161 (4.88)	14,569 (3.72)	70,704	43,454 (5.46)	27,250 (6.04)

© 2024 Park E et al. *JAMA Network Open*

eTable 2. General Characteristics of Study Participants and the Excluded Participants by Gender in 2002 and 2003

	Study participants (remaining)		Excluded participants	
	Men	Women	Men	Women
Characteristic				
Total, n	1,727,340	1,247,480	2,761,269	1,601,966
Person-years, years	23,026,715	16,705,859	35,279,557	20,345,639
All cancer, n (%)	117,805 (6.82)	73,976 (5.93)	326,458 (11.82)	188,407 (11.76)
Lung cancer, n (%)	16,928 (0.98)	6,237 (0.50)	45,753 (1.66)	14,438 (0.90)
Liver cance, n (%)	9,846 (0.57)	2,620 (0.21)	33,736 (1.22)	8,490 (0.53)
Stomach cancer, n (%)	22,628 (1.31)	7,983 (0.64)	66,914 (2.42)	21,855 (1.36)
Colorectal cancer, n (%)	15,201 (0.88)	7,985 (0.64)	48,682 (1.76)	22,831 (1.43)
Age, n (%)				
30 to 39	730,321 (42.28)	211,626 (16.96)	978,632 (35.44)	241,664 (15.09)
40 to 49	566,559 (32.80)	482,608 (38.69)	852,643 (30.88)	530,206 (33.10)
50 to 59	287,173 (16.63)	348,416 (27.93)	477,867 (17.31)	323,781 (20.21)
60 to 69	126,479 (7.32)	182,177 (14.60)	323,201 (11.70)	327,250 (20.43)
70 or more	16,808 (0.97)	22,653 (1.82)	128,926 (4.67)	179,065 (11.18)
Family income, n (%)				
Medical aid or 1st quartile	171,055 (9.90)	319,614 (25.62)	381,923 (13.83)	419,068 (26.16)
2nd quartile	346,561 (20.06)	245,285 (19.66)	576,971 (20.90)	337,825 (21.09)
3rd quartile	568,572 (32.92)	297,497 (23.85)	769,045 (27.85)	348,109 (21.73)
4th quartile	641,152 (37.12)	385,084 (30.87)	928,469 (33.62)	463,587 (28.94)
missing	-	-	104,861 (3.80)	33,377 (2.08)
Smoking status (at baseline), n (%)				
Never smokers	657,870 (38.09)	1,226,518 (98.32)	870,724 (31.53)	1,417,754 (88.50)
Past smokers	-	-	712,150 (25.79)	35,310 (2.20)
Current smokers	1,069,470 (61.91)	20,962 (1.68)	1,124,330 (40.72)	57,210 (3.57)
missing	-	-	54,065 (1.96)	91,692 (5.72)
Alcohol consumption, n (%)				
Non	496,368 (28.74)	1,005,646 (80.61)	861,814 (31.21)	1,215,488 (75.87)
< 24g per day	1,027,886 (59.51)	235,246 (18.86)	1,461,209 (52.92)	294,680 (18.39)
>= 24g per day	203,086 (11.76)	6,588 (0.53)	360,317 (13.05)	12,494 (0.78)
missing	-	-	77,929 (2.82)	79,304 (4.95)
BMI, kg/m^2, n (%)				
<23	640,458 (37.08)	568,839 (45.60)	973,619 (35.26)	707,038 (44.14)
23-24.9	484,609 (28.06)	311,551 (24.97)	752,532 (27.25)	378,195 (23.61)
>=25	602,273 (34.87)	367,090 (29.43)	1,031,899 (37.37)	513,689 (32.07)
missing	-	-	3,219 (0.12)	3,044 (0.19)

© 2024 Park E et al. *JAMA Network Open*

Physical activity, n (%)				
No	807,127 (46.73)	830,560 (66.58)	1,281,482 (46.41)	1,045,856 (65.29)
1-2 days per week	606,864 (35.13)	219,581 (17.60)	819,456 (29.68)	243,102 (15.18)
3-4 days per week	191,497 (11.09)	98,237 (7.87)	287,241 (10.40)	102,397 (6.39)
5-6 days per week	42,122 (2.44)	28,398 (2.28)	68,060 (2.46)	31,045 (1.94)
7 days per week	79,730 (4.62)	70,704 (5.67)	147,654 (5.35)	91,849 (5.73)
missing			157,376 (5.70)	87,717 (5.48)

© 2024 Park E et al. *JAMA Network Open*

eTable 3. Hazard Ratios of Cancer Incidence by Time-Updated Smoking Status Among Women

Time-updated smoking status	Participants, No.	Incident cancer cases, No.	Person-years	Unadjusted HRs (95% CI)	Unadjusted *P*-value	Age-adjusted HRs (95% CI)	Age-adjusted *P*-value	Multi-variable-adjusted HRs (95% CI)	Multi-variable-adjusted *P*-value
**All sites**									
Continuous smokers	2 704	225	36 142	1 [Reference]		1 [Reference]		1 [Reference]	
Relapsed smokers	3 834	324	51 205	1.02 (0.73, 1.43)	.93	0.95 (0.68, 1.34)	.78	1.00 (0.71, 1.40)	.98
Transient quitters	3 619	332	48 274	1.11 (0.79, 1.55)	.56	1.01 (0.72, 1.42)	.94	1.03 (0.73, 1.45)	.88
Complete quitters	10 805	775	144 424	0.86 (0.64, 1.16)	.32	0.82 (0.61, 1.11)	.20	0.86 (0.64, 1.17)	.34
Never smokers	1 226 518	72 290	16 425 814	0.70 (0.54, 0.91)	.008	0.70 (0.54, 0.90)	.006	0.56 (0.39, 0.79)	.001
P for trend				<.001		<.001		.21	
**Lung**									
Continuous smokers	2 704	45	36 142	1 [Reference]		1 [Reference]		1 [Reference]	
Relapsed smokers	3 834	70	51 205	1.11 (0.57, 2.17)	.76	0.96 (0.49, 1.89)	.92	0.95 (0.48, 1.88)	.89
Transient quitters	3 619	57	48 274	0.97 (0.48, 1.94)	.92	0.81 (0.40, 1.62)	.54	0.83 (0.41, 1.69)	.60
Complete quitters	10 805	111	144 424	0.63 (0.34, 1.16)	.14	0.57 (0.31, 1.06)	.08	0.64 (0.34, 1.19)	.16
Never smokers	1 226 518	5 954	16 425 814	0.29 (0.17, 0.50)	<.001	0.29 (0.17, 0.48)	<.001	0.13 (0.07, 0.24)	<.001
P for trend				<.001		<.001		.08	
**Colon & rectum**									
Continuous smokers	2 704	30	36 142	1 [Reference]		1 [Reference]		1 [Reference]	
Relapsed smokers	3 834	30	51 205	0.71 (0.25, 2.01)	.52	0.60 (0.21, 1.70)	.33	0.64 (0.22, 1.83)	.40
Transient quitters	3 619	38	48 274	0.97 (0.36, 2.60)	.94	0.77 (0.29, 2.07)	.60	0.86 (0.32, 2.33)	.77
Complete quitters	10 805	89	144 424	0.75 (0.32, 1.77)	.51	0.67 (0.28, 1.57)	.35	0.73 (0.31, 1.74)	.48
Never smokers	1 226 518	7 798	16 425 814	0.58 (0.27, 1.21)	.15	0.56 (0.27, 1.17)	.12	0.52 (0.19, 1.38)	.19
P for trend				.03		.09		.69	

Hazard ratios and 95% confidence intervals were adjusted for age (in year, continuous and quadratic terms), body-mass index (in continuous term), income level (Medical aid or 1st quartile, 2nd quartile, 3rd quartile, or 4th quartile), physical activity (no, 1-2 days per week, 3-4 days per week, 5-6 days per week, or 7 days per week), alcohol consumption (Non-drinkers, <24g per day, or ≥24g per day), and pack-years (never smokers, <10 pack-years, 10 to <20 pack-years, or ≥20 pack-years). Hazard ratios for liver cancer were further adjusted for chronic viral hepatitis (negative or positive) and liver cirrhosis (negative or positive).

© 2024 Park E et al. *JAMA Network Open*

eTable 4. General Characteristics of Study Participants Who Underwent Health Screening From 2006 to 2008

	Men (N=3 278 144)			Women (N=2 007 845)		
	Current smokers	Past smokers	Never smokers	Current smokers	Past smokers	Never smokers
Characteristic	N (%)	N (%)	N (%)	N (%)	N (%)	N (%)
Total	1 291 833	433 922	1 552 389	34 158	11 429	1 962 258
Age						
30 to 39	396 128 (30.7)	88 592 (20.4)	305 551 (19.7)	2 575 (7.5)	1 266 (11.1)	187 109 (9.5)
40 to 49	474 050 (36.7)	149 157 (34.4)	483 135 (31.1)	8 644 (25.3)	3 147 (27.5)	556 884 (28.4)
50 to 59	268 319 (20.8)	111 449 (25.7)	392 766 (25.3)	10 210 (29.9)	3 618 (31.7)	610 291 (31.1)
60 to 69	110 640 (8.6)	56 219 (13.0)	239 628 (15.4)	6 237 (18.3)	1 806 (15.8)	385 833 (19.7)
70+	42 696 (3.3)	28 505 (6.6)	131 309 (8.5)	6 492 (19.0)	1 592 (13.9)	222 141 (11.3)
Income level						
Medical aid or 1st quartile	139 486 (10.8)	36 574 (8.4)	168 761 (10.9)	9 150 (26.8)	2 253 (19.7)	424 244 (21.6)
2nd quartile	208 858 (16.2)	52 863 (12.2)	223 562 (14.4)	8 566 (25.1)	2 280 (20.0)	369 624 (18.8)
3rd quartile	410 910 (31.8)	114 501 (26.4)	425 703 (27.4)	8 512 (24.9)	3 018 (26.4)	476 632 (24.3)
4th quartile	532 579 (41.2)	229 984 (53.0)	734 363 (47.3)	7 930 (23.2)	3 878 (33.9)	691 758 (35.3)
Alcohol consumption						
Non-drinkers	251 845 (19.5)	96 683 (22.3)	805 762 (51.9)	17 620 (51.6)	5 042 (44.1)	1 627 489 (82.9)
< 24g per day	812 145 (62.9)	276 532 (63.7)	641 423 (41.3)	14 053 (41.1)	6 016 (52.6)	326 043 (16.6)
≥ 24g per day	227 843 (17.6)	60 707 (14.0)	105 204 (6.8)	2 485 (7.3)	371 (3.3)	8 726 (0.4)
BMI						
<23 kg/m2	480 164 (37.2)	125 648 (29.0)	505 993 (32.6)	16 749 (49.0)	5 126 (44.9)	872 164 (44.5)
23 to <25	352 550 (27.3)	132 273 (30.5)	458 456 (29.5)	7 626 (22.3)	2 768 (24.2)	492 801 (25.1)
≥25	459 119 (35.5)	176 001 (40.6)	587 940 (37.9)	9 783 (28.6)	3 535 (30.9)	597 293 (30.4)
Physical activity						
No	553 294 (42.8)	140 891 (32.5)	722 644 (46.6)	21 420 (62.7)	4 959 (43.4)	1 153 727 (58.8)
1-2 days per week	502 278 (38.9)	164 390 (37.9)	477 734 (30.8)	6 506 (19.1)	4 242 (37.1)	410 247 (20.9)
3-4 days per week	153 628 (11.9)	81 604 (18.8)	211 941 (13.7)	3 081 (9.0)	1 215 (10.6)	217 456 (11.1)
5-6 days per week	29 853 (2.3)	19 351 (4.5)	46 888 (3.0)	946 (2.8)	422 (3.7)	58 074 (3.0)
7 days per week	52 780 (4.1)	27 686 (6.4)	93 182 (6.0)	2 205 (6.5)	591 (5.2)	122 754 (6.3)
Chronic viral hepatitis						
Negative	1 131 229 (87.6)	369 105 (85.1)	1 319 466 (85.0)	29 131 (85.3)	9 707 (84.9)	1 692 302 (86.2)
Positive	160 604 (12.4)	64 817 (14.9)	232 923 (15.0)	5 027 (14.7)	1 722 (15.1)	269 956 (13.8)
Liver cirrhosis						
Negative	1 289 254 (99.8)	433 433 (99.9)	1 550 200 (99.9)	34 126 (99.9)	11 425 (100.0)	1 962 072 (100.0)
Positive	2 579 (0.2)	489 (0.1)	2 189 (0.1)	32 (0.1)	4 (0.0)	186 (0.0)

Information was collected from baseline (2006-2008)

© 2024 Park E et al. *JAMA Network Open*

eTable 5. Hazard Ratios of Cancer Incidence Among Male Participants Who Underwent Health Screening From 2006 to 2008 by Self-Reported Smoking Status

				Unadjusted		Age-adjusted		Multi-variable-adjusted	
Self-reported smoking status	Participants, No.	Incident cancer cases, No.	Person-years	HRs (95% CI)	P-value	HRs (95% CI)	P-value	HRs (95% CI)	P-value
All cancer									
Current smokers	1 291 833	102 809	18 392 904	1 [Reference]		1 [Reference]		1 [Reference]	
Past smokers	433 922	36 393	6 160 018	1.06 (1.05, 1.07)	<.001	0.80 (0.79, 0.81)	<.001	0.80 (0.79, 0.81)	<.001
Never smokers	1 552 389	134 001	21 915 934	1.10 (1.09, 1.11)	<.001	0.75 (0.74, 0.75)	<.001	0.75 (0.75, 0.76)	<.001
Lung cancer									
Current smokers	1 291 833	19 334	18 392 904	1 [Reference]		1 [Reference]		1 [Reference]	
Past smokers	433 922	4 340	6 160 018	0.67 (0.65, 0.69)	<.001	0.44 (0.42, 0.45)	<.001	0.47 (0.46, 0.49)	<.001
Never smokers	1 552 389	14 146	21 915 934	0.62 (0.60, 0.63)	<.001	0.34 (0.33, 0.35)	<.001	0.35 (0.34, 0.35)	<.001
Liver cancer									
Current smokers	1 291 833	9 767	18 392 904	1 [Reference]		1 [Reference]		1 [Reference]	
Past smokers	433 922	2 892	6 160 018	0.89 (0.85, 0.92)	<.001	0.69 (0.66, 0.71)	<.001	0.68 (0.66, 0.71)	<.001
Never smokers	1 552 389	11 799	21 915 934	1.02 (0.99, 1.04)	.26	0.72 (0.70, 0.74)	<.001	0.71 (0.69, 0.73)	<.001
Stomach cancer									
Current smokers	1 291 833	20 921	18 392 904	1 [Reference]		1 [Reference]		1 [Reference]	
Past smokers	433 922	7 198	6 160 018	1.03 (1.00, 1.06)	.04	0.79 (0.77, 0.81)	<.001	0.80 (0.78, 0.82)	<.001
Never smokers	1 552 389	25 584	21 915 934	1.03 (1.01, 1.05)	.003	0.72 (0.71, 0.74)	<.001	0.74 (0.72, 0.75)	<.001
Colorectal cancer									
Current smokers	1 291 833	13 687	18 392 904	1 [Reference]		1 [Reference]		1 [Reference]	
Past smokers	433 922	5 314	6 160 018	1.16 (1.12, 1.20)	<.001	0.88 (0.85, 0.91)	<.001	0.90 (0.87, 0.93)	<.001
Never smokers	1 552 389	19 248	21 915 934	1.18 (1.16, 1.21)	<.001	0.82 (0.80, 0.84)	<.001	0.86 (0.84, 0.88)	<.001

Hazard ratios and 95% confidence intervals were adjusted for age (in year, continuous and quadratic terms), body-mass index (in continuous term), income level (medical aid or 1st quartile, 2nd quartile, 3rd quartile, or 4th quartile), physical activity (no, 1-2 days per week, 3-4 days per week, 5-6 days per week, or 7 days per week), and alcohol consumption (non-drinkers, <24g per day, or ≥24g per day). Hazard ratios for liver cancer were further adjusted for chronic viral hepatitis (negative or positive) and liver cirrhosis (negative or positive).

© 2024 Park E et al. *JAMA Network Open*

eTable 6. Hazard Ratios of Cancer Incidence Among Female Participants Who Underwent Health Screening From 2006 to 2008 by Self-Reported Smoking Status

				Unadjusted		Age-adjusted		Multi-variable-adjusted	
**Self-reported smoking status**	**Participants, No.**	**Incident cancer cases, No.**	**Person-years**	**HRs (95% CI)**	**P-value**	**HRs (95% CI)**	**P-value**	**HRs (95% CI)**	**P-value**
All cancer									
Current smokers	34 158	3 253	469 615	1 [Reference]		1 [Reference]		1 [Reference]	
Past smokers	11 429	911	160 553	0.82 (0.76, 0.88)	<.001	0.85 (0.79, 0.91)	<.001	0.83 (0.77, 0.89)	<.001
Never smokers	1 962 258	150 299	27 889 923	0.77 (0.75, 0.80)	<.001	0.81 (0.78, 0.84)	<.001	0.79 (0.76, 0.82)	<.001
P for trend									
Lung cancer									
Current smokers	34 158	652	469 615	1 [Reference]		1 [Reference]		1 [Reference]	
Past smokers	11 429	102	160 553	0.45 (0.37, 0.56)	<.001	0.51 (0.41, 0.63)	<.001	0.52 (0.42, 0.64)	<.001
Never smokers	1 962 258	11 643	27 889 923	0.30 (0.28, 0.32)	<.001	0.34 (0.31, 0.36)	<.001	0.33 (0.30, 0.36)	<.001
Liver cancer									
Current smokers	34 158	177	469 615	1 [Reference]		1 [Reference]		1 [Reference]	
Past smokers	11 429	39	160 553	0.64 (0.45, 0.91)	.01	0.73 (0.52, 1.03)	.08	0.74 (0.52, 1.05)	.09
Never smokers	1 962 258	5 739	27 889 923	0.54 (0.47, 0.63)	<.001	0.61 (0.53, 0.71)	<.001	0.62 (0.53, 0.72)	<.001
Stomach cancer									
Current smokers	34 158	363	469 615	1 [Reference]		1 [Reference]		1 [Reference]	
Past smokers	11 429	95	160 553	0.76 (0.61, 0.96)	.02	0.84 (0.67, 1.06)	.13	0.84 (0.67, 1.06)	.14
Never smokers	1 962 258	16 532	27 889 923	0.76 (0.69, 0.85)	<.001	0.85 (0.76, 0.94)	.002	0.86 (0.77, 0.95)	.004
Colorectal cancer									
Current smokers	34 158	384	469 615	1 [Reference]		1 [Reference]		1 [Reference]	
Past smokers	11 429	107	160 553	0.81 (0.66, 1.01)	.06	0.91 (0.73, 1.12)	.37	0.90 (0.72, 1.11)	.32
Never smokers	1 962 258	17 069	27 889 923	0.74 (0.67, 0.82)	<.001	0.84 (0.76, 0.92)	.001	0.85 (0.76, 0.94)	.001

Hazard ratios and 95% confidence intervals were adjusted for age (in year, continuous and quadratic terms), body-mass index (in continuous term), income level (medical aid or 1st quartile, 2nd quartile, 3rd quartile, or 4th quartile), physical activity (no, 1-2 days per week, 3-4 days per week, 5-6 days per week, or 7 days per week), and alcohol consumption (non-drinkers, <24g per day, or ≥24g per day). Hazard ratios for liver cancer were further adjusted for chronic viral hepatitis (negative or positive) and liver cirrhosis (negative or positive).

© 2024 Park E et al. *JAMA Network Open*

eTable 6. Hazard Ratios of Cancer Incidence Among Female Participants Who Underwent Health Screening From 2006 to 2008 by Self-Reported Smoking Status (continued)

				Unadjusted		Age-adjusted		Multi-variable-adjusted	
**Years since quitting**	**Participants, No.**	**Incident cancer cases, No.**	**Person-years**	**HRs (95% CI)**	**P-value**	**HRs (95% CI)**	**P-value**	**HRs (95% CI)**	**P-value**
**All cancer**									
Current smokers	1 291 833	102 809	18 392 904	1 [Reference]		1 [Reference]		1 [Reference]	
<10 years	105 510	8 029	1 507 766	0.95 (0.93, 0.97)	<.001	0.84 (0.83, 0.86)	<.001	0.85 (0.83, 0.86)	<.001
10 to <15 years	19 265	1 807	271 015	1.20 (1.14, 1.25)	<.001	0.80 (0.76, 0.84)	<.001	0.80 (0.77, 0.84)	<.001
15 to <20 years	10 099	985	141 875	1.25 (1.17, 1.33)	<.001	0.77 (0.72, 0.82)	<.001	0.78 (0.73, 0.83)	<.001
≥20 years	7 256	813	99 694	1.47 (1.38, 1.58)	<.001	0.70 (0.65, 0.75)	<.001	0.70 (0.66, 0.75)	<.001
Never smokers	1 552 389	134 001	21 915 934	1.10 (1.09, 1.11)	<.001	0.75 (0.74, 0.75)	<.001	0.75 (0.75, 0.76)	<.001
**Lung cancer**									
Current smokers	1 291 833	19 334	18 392 904	1 [Reference]		1 [Reference]		1 [Reference]	
<10 years	105 510	1 022	1 507 766	0.64 (0.61, 0.69)	<.001	0.54 (0.50, 0.57)	<.001	0.57 (0.54, 0.61)	<.001
10 to <15 years	19 265	224	271 015	0.79 (0.69, 0.90)	<.001	0.44 (0.38, 0.50)	<.001	0.47 (0.41, 0.53)	<.001
15 to <20 years	10 099	101	141 875	0.68 (0.56, 0.83)	<.001	0.34 (0.28, 0.42)	<.001	0.37 (0.30, 0.45)	<.001
≥20 years	7 256	98	99 694	0.95 (0.78, 1.15)	.58	0.33 (0.27, 0.40)	<.001	0.35 (0.29, 0.43)	<.001
Never smokers	1 552 389	14 146	21 915 934	0.62 (0.60, 0.63)	<.001	0.34 (0.33, 0.35)	<.001	0.35 (0.34, 0.35)	<.001
**Liver cancer**									
Current smokers	1 291 833	9 767	18 392 904	1 [Reference]		1 [Reference]		1 [Reference]	
<10 years	105 510	608	1 507 766	0.76 (0.70, 0.82)	<.001	0.68 (0.63, 0.74)	<.001	0.67 (0.62, 0.73)	<.001
10 to <15 years	19 265	153	271 015	1.07 (0.91, 1.25)	.44	0.74 (0.63, 0.87)	<.001	0.73 (0.62, 0.85)	<.001
15 to <20 years	10 099	85	141 875	1.13 (0.91, 1.40)	.26	0.73 (0.59, 0.90)	.003	0.74 (0.60, 0.91)	.005
≥20 years	7 256	67	99 694	1.27 (1.00, 1.62)	.05	0.65 (0.51, 0.83)	.001	0.65 (0.51, 0.82)	<.001
Never smokers	1 552 389	11 799	21 915 934	1.02 (0.99, 1.04)	.26	0.73 (0.71, 0.75)	<.001	0.71 (0.69, 0.73)	<.001

© 2024 Park E et al. *JAMA Network Open*

eTable 7. Hazard Ratios of Cancer Incidence Among Male Participants Who Underwent Health Screening from 2006 to 2008 by Categorized Years Since Quitting

				Unadjusted		Age-adjusted		Multi-variable-adjusted	
**Years since quitting**	**Participants, No.**	**Incident cancer cases, No.**	**Person-years**	**HRs (95% CI)**	**P-value**	**HRs (95% CI)**	**P-value**	**HRs (95% CI)**	**P-value**
**Stomach cancer**									
Current smokers	1 291 833	20 921	18 392 904	1 [Reference]		1 [Reference]		1 [Reference]	
<10 years	105 510	1 629	1 507 766	0.95 (0.90, 1.00)	.04	0.85 (0.81, 0.89)	<.001	0.86 (0.81, 0.90)	<.001
10 to <15 years	19 265	344	271 015	1.12 (1.01, 1.24)	.04	0.77 (0.69, 0.85)	<.001	0.78 (0.70, 0.86)	<.001
15 to <20 years	10 099	198	141 875	1.23 (1.07, 1.42)	.004	0.78 (0.68, 0.89)	<.001	0.79 (0.69, 0.91)	.001
≥20 years	7 256	166	99 694	1.48 (1.27, 1.72)	<.001	0.73 (0.63, 0.86)	<.001	0.75 (0.64, 0.87)	<.001
Never smokers	1 552 389	25 584	21 915 934	1.03 (1.01, 1.05)	.003	0.72 (0.71, 0.74)	<.001	0.74 (0.72, 0.75)	<.001
**Colorectal cancer**									
Current smokers	1 291 833	13 687	18 392 904	1 [Reference]		1 [Reference]		1 [Reference]	
<10 years	105 510	1 199	1 507 766	1.07 (1.01, 1.13)	.03	0.95 (0.90, 1.01)	.10	0.96 (0.91, 1.02)	.20
10 to <15 years	19 265	254	271 015	1.26 (1.11, 1.43)	<.001	0.85 (0.75, 0.96)	.01	0.87 (0.77, 0.99)	.03
15 to <20 years	10 099	133	141 875	1.26 (1.06, 1.50)	.008	0.79 (0.66, 0.93)	.006	0.81 (0.69, 0.96)	.02
≥20 years	7 256	122	99 694	1.66 (1.39, 1.98)	<.001	0.80 (0.67, 0.96)	.02	0.83 (0.69, 0.99)	.03
Never smokers	1 552 389	19 248	21 915 934	1.18 (1.16, 1.21)	<.001	0.82 (0.80, 0.84)	<.001	0.86 (0.84, 0.88)	<.001

Hazard ratios and 95% confidence intervals were adjusted for age (in year, continuous and quadratic terms), body-mass index (in continuous term), income level (medical aid or 1st quartile, 2nd quartile, 3rd quartile, or 4th quartile), physical activity (no, 1-2 days per week, 3-4 days per week, 5-6 days per week, or 7 days per week), and alcohol consumption (non-drinkers, <24g per day, or ≥24g per day). Hazard ratios for liver cancer were further adjusted for chronic viral hepatitis (negative or positive) and liver cirrhosis (negative or positive).

© 2024 Park E et al. *JAMA Network Open*

eFigure 1. Study Population Flowchart

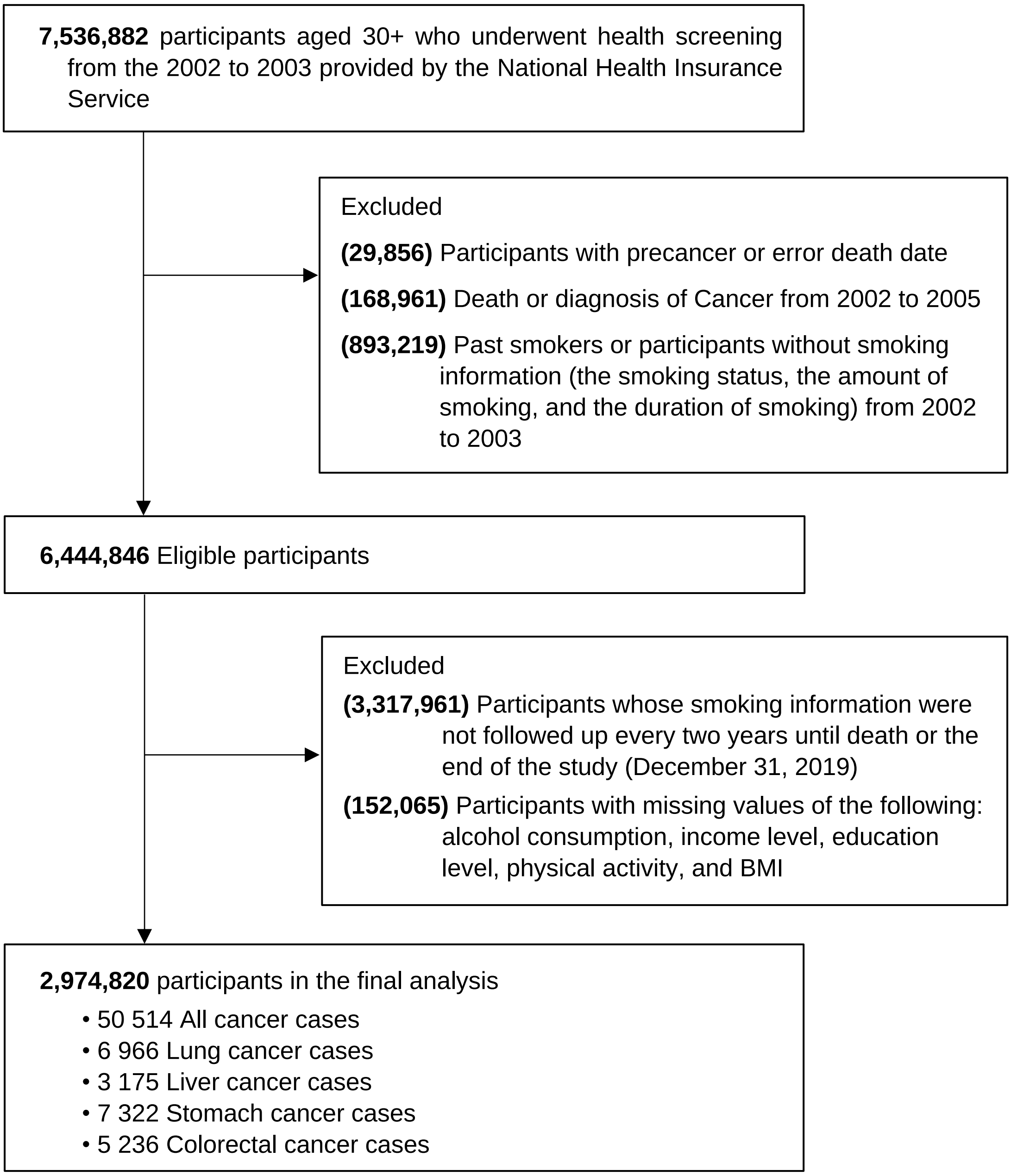

© 2024 Park E et al. *JAMA Network Open*

eFigure 2. Definition of Time-Updated Smoking Status and Duration of Smoking Cessation During Follow-Up (2009-2019)

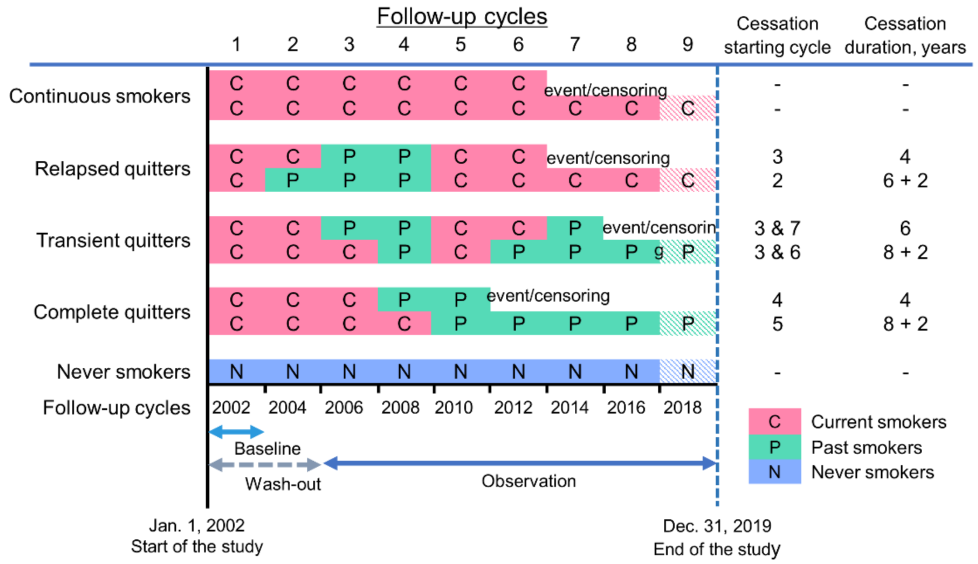

The letter C indicates current smokers, P for past smokers, and N for never smokers.

Smoking behavior from 2018 to 2019 was regarded as smoking behavior of last cycle.

eFigure 3. Cancer Incidence Rates per 100,000 Persons in Male Participants at Each Time Point From 2006 to 2019

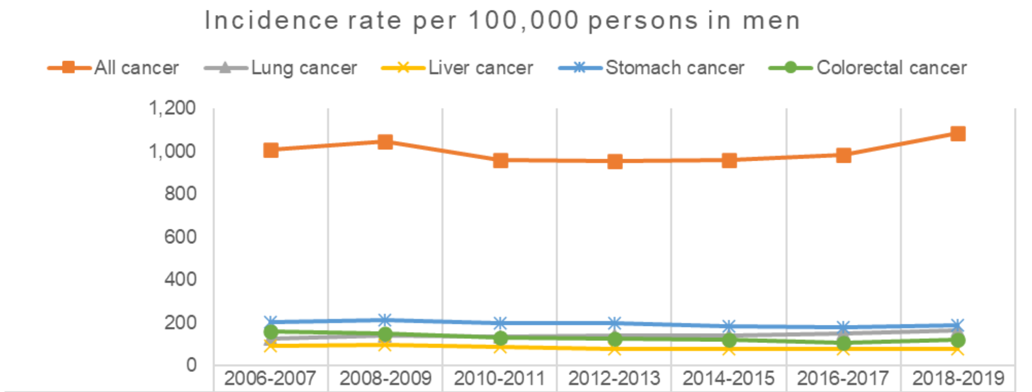

	2006-2007	2008-2009	2010-2011	2012-2013	2014-2015	2016-2017	2018-2019
All cancer	1,011	1,048	959	956	961	985	1,084
Lung cancer	126	139	136	138	140	147	163
Liver cancer	91	94	86	78	78	77	79
Stomach cancer	202	211	197	198	182	179	186
Colorectal cancer	160	151	129	123	120	108	119

© 2024 Park E et al. *JAMA Network Open*

eFigure 4. Cancer Incidence Rates per 100,000 Persons in Female Participants at Each Time Point From 2006 to 2019

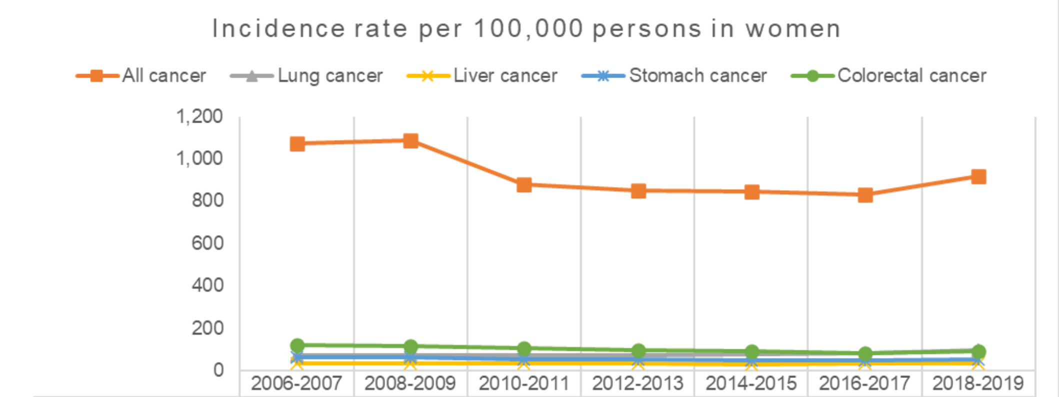

	2006-2007	2008-2009	2010-2011	2012-2013	2014-2015	2016-2017	2018-2019
All cancer	1,074	1,089	881	852	845	831	919
Lung cancer	71	72	70	71	77	81	97
Liver cancer	34	34	33	32	30	32	33
Stomach cancer	62	65	51	53	47	46	54
Colorectal cancer	121	115	105	96	92	84	93

© 2024 Park E et al. *JAMA Network Open*

eFigure 5. Study Population Flowchart for Sensitivity Analysis

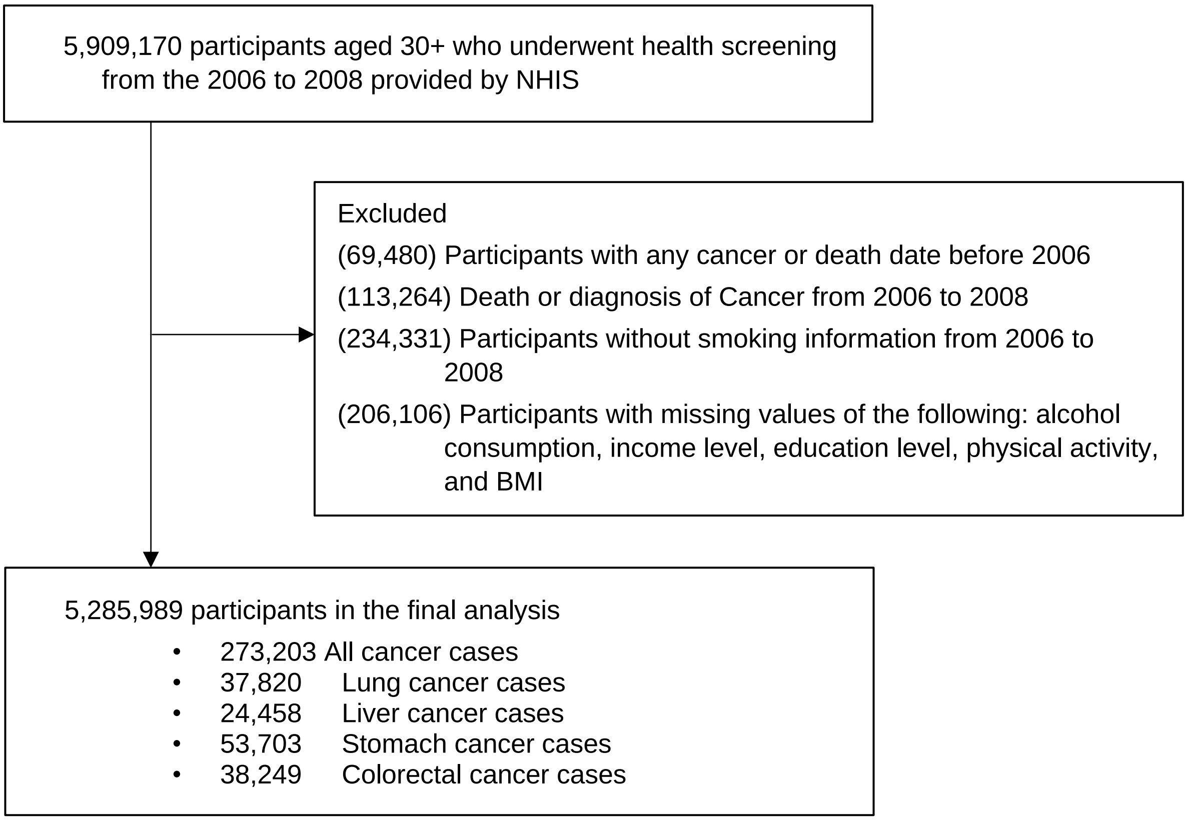

© 2024 Park E et al. *JAMA Network Open*

eFigure 6. Definition of Smoking Status and Duration of Smoking Cessation in the Sensitivity Analysis

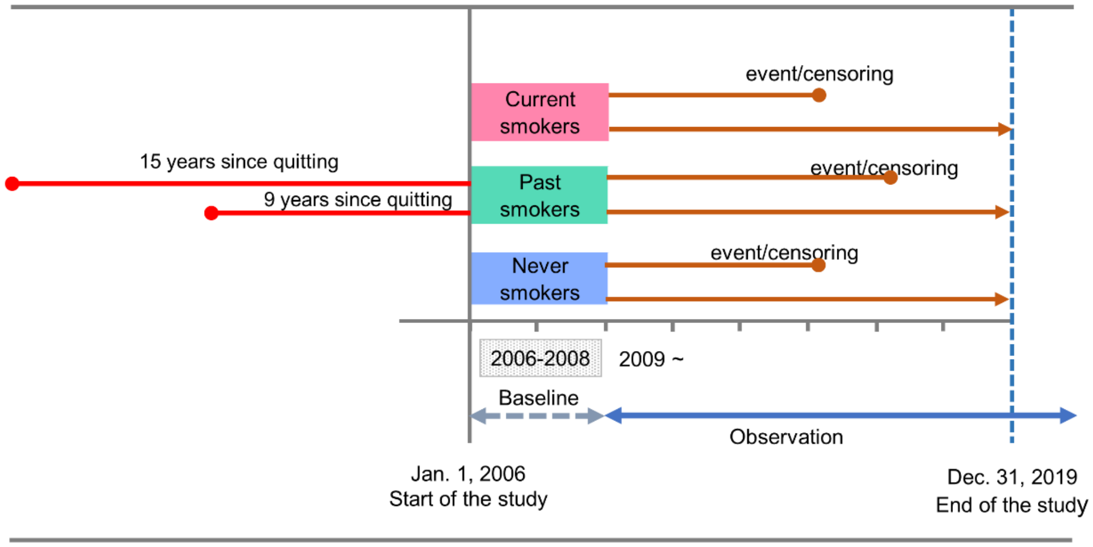

© 2024 Park E et al. *JAMA Network Open*

eFigure 7. Hazard Ratio and 95% Confidence Intervals of Self-Reported Past Smokers Compared to Current Smokers According to Years Since Quitting Among Men Who Underwent Health Screening From 2006 to 2008

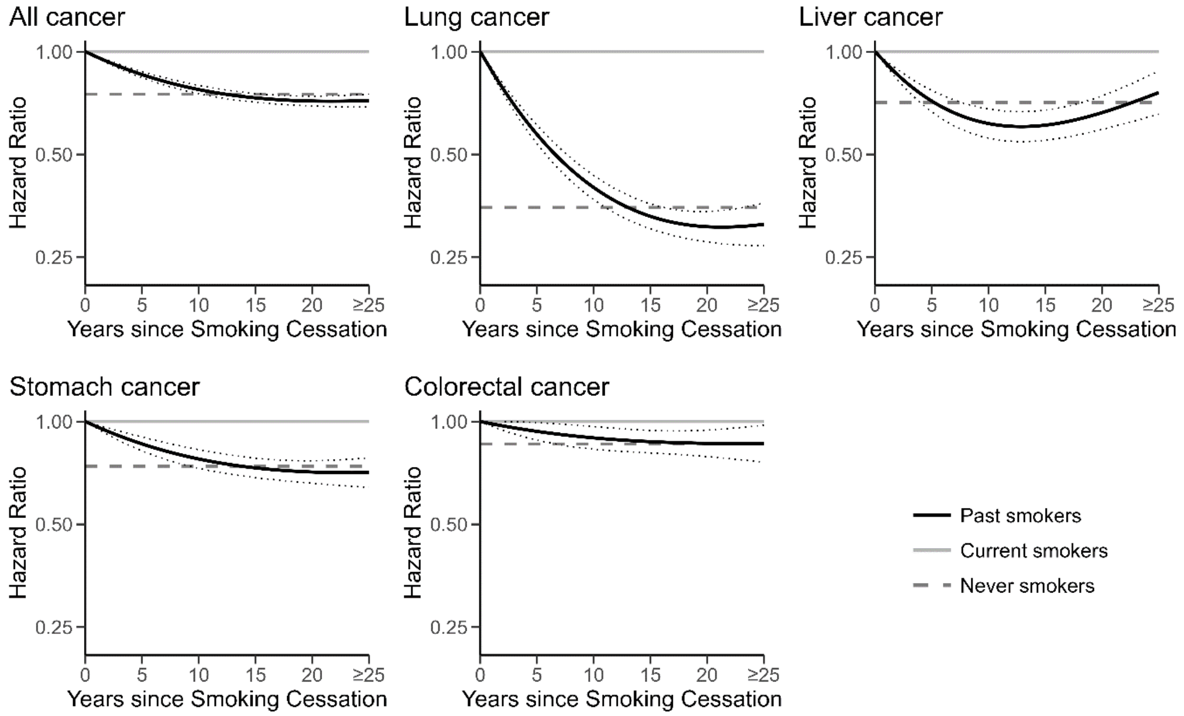

Hazard ratios and 95% confidence intervals were adjusted for age (in year, continuous and quadratic terms), body-mass index (in continuous term), income level (medical aid/1st quartile, 2nd quartile, 3rd quartile, or 4th quartile), physical activity (no, 1-2 days per week, 3-4 days per week, 5-6 days per week, or 7 days per week), and alcohol consumption (non-drinkers, <24g per day, or ≥24g per day). Hazard ratios for liver cancer were further adjusted for chronic viral hepatitis (negative or positive) and liver cirrhosis (negative or positive). Dotted lines represent 95% confidence intervals. Splines have 4 knots at 5th, 35th, 65th, and 95th percentiles of years since quitting for all cancer, stomach cancer, and liver cancer; Splines have 5 knots at 5th, 27.5th 50th, 72.5th, and 95th percentiles of them for lung cancer and colorectal cancer.

© 2024 Park E et al. *JAMA Network Open*

eFigure 8. Hazard Ratio and 95% Confidence Intervals of Self-Reported Past Smokers Compared to Current Smokers According to Years Since Quitting Among Women Who Underwent Health Screening From 2006 to 2008

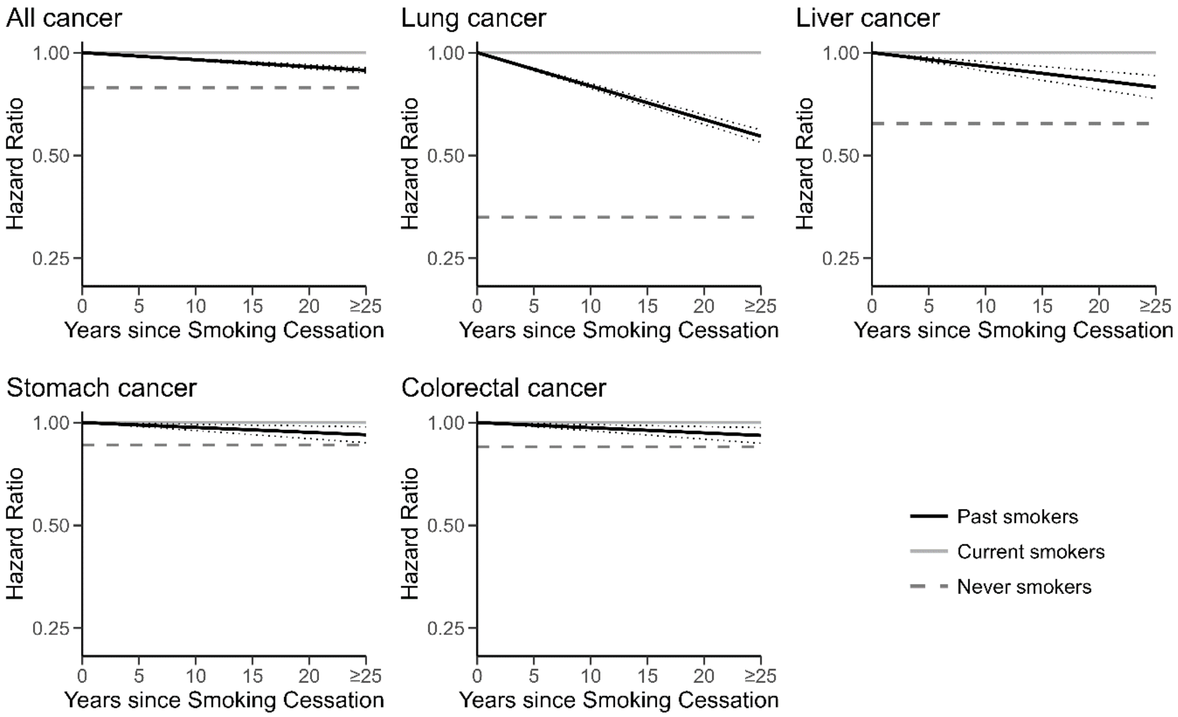

Hazard ratios and 95% confidence intervals were adjusted for age (in year, continuous and quadratic terms), body-mass index (in continuous term), income level (medical aid/1st quartile, 2nd quartile, 3rd quartile, or 4th quartile), physical activity (no, 1-2 days per week, 3-4 days per week, 5-6 days per week, or 7 days per week), and alcohol consumption (non-drinkers, <24g per day, or ≥24g per day). Hazard ratios for liver cancer were further adjusted for chronic viral hepatitis (negative or positive) and liver cirrhosis (negative or positive). Dotted lines represent 95% confidence intervals. Splines have 3 knots at 10th, 50th, and 90th percentiles of years since quitting for all cancer, lung cancer, stomach cancer, and liver cancer; Splines have 4 knots at 5th, 35th, 65th, and 95th percentiles of them for colorectal cancer.

© 2024 Park E et al. *JAMA Network Open*
