# Supplementary material for: Cancer Risk Following Smoking Cessation in Korea
Source: JAMA Netw Open. 2024 Feb 6;7(2):e2354958. doi: 10.1001/jamanetworkopen.2023.54958 (PMC10848071; doi:10.1001/jamanetworkopen.2023.54958)
Supplement: Supplement 2. — Data Sharing Statement [file jamanetwopen-e2354958-s002.pdf]

Data Sharing Statement

Park. Cancer Risk Following Smoking Cessation in Korea. *JAMA Netw Open*. Published February 06, 2024. doi:10.1001/jamanetworkopen.2023.54958

Data
Data available: No

Additional Information
Explanation for why data not available: The data used in this study (NHIS-2022-1-785) were provided by the National Health Insurance Service. To protect personal information, data cannot be shared because the NHIS prohibits the transfer, rental, or sale of the database to third parties, except for researchers who have been approved for access. The NHIS data can be requested through the NHIS website (https://nhiss.nhis.or.kr).
